# Supplementary material for: High-throughput immune repertoire analysis with IGoR
Source: Nat Commun. 2018 Feb 8;9:561. doi: 10.1038/s41467-018-02832-w (PMC5805751; doi:10.1038/s41467-018-02832-w)
Supplement: Supplementary file 1 — Supplementary Information [file 41467_2018_2832_MOESM1_ESM.pdf]

IGoR: a tool for high-throughput immune repertoire analysis — Supplementary Information

Marcou et al.

Supplementary Note 1 – Model definitions

We start by giving the particular model structures used in this study. We then give a more general definition applicable to other general types of recombination products.

a. Models for TRA, TRB and IGH

We define a probabilistic model for each type of chain (e.g. α, β, heavy, light) that describes the probability of each recombination event $\boldsymbol{E}$ by the probabilities of the known elements of the recombination subprocess (gene choice, insertions, deletions at each of the junctions etc) for each chain, and assumes only the minimum correlations between the subprocesses needed to explain the correlations observed in the data. We model insertions as a Markov chain (the identity of an inserted nucleotide only depends on the previously inserted one) with a nonparametric length distribution [1–3]. For each insertion site (X= VD and DJ for β and heavy chains and X=VJ for α and light chains) we infer the probability of observing a non-templated sequence of a given length, $P(\mathrm{insX})$, and the transition matrices $P_{\mathrm{VJ}}(n_i|n_{i-1})$, $P_{\mathrm{VD}}(n_i|n_{i-1})$, $P_{\mathrm{DJ}}(m_i|m_{i-1})$ giving the probability of inserting a given nucleotide as a function of the identity of previous one. For each gene we infer the probability of the number of deletions conditioned on the gene identity, e.g. $P(\mathrm{del}V|V)$ for deletions from the V gene. We model templated palindromic insertions as negative deletions [1, 2]. The D gene is very short and may get fully deleted. This introduces correlations between the deletions on both sides of the original D gene template. We account for these correlations by inferring the joint probability $P(\mathrm{del}Dl, \mathrm{del}Dr|D)$. We treat every allele as a different gene [2] and infer the joint gene usage $P(V, D, J)$ for β and heavy chains, and $P(V, J)$ for α and light chains, to be able to capture correlations between segment usage.

For TCR α chains or BCR light chains, the probability of a recombination event $\boldsymbol{E} = (V, J, \mathrm{del}V, \mathrm{del}J, \mathrm{insVJ})$ is:

$$P^{\alpha/\mathrm{L}}_{\mathrm{recomb}}(\boldsymbol{E}) = P(V,J)P(\mathrm{del}V|V)P(\mathrm{del}J|J) \times P(\mathrm{insVJ}) \prod_{i}^{\mathrm{insVJ}} P_{\mathrm{VJ}}(n_i|n_{i-1}) \tag{1}$$

Similarly, the probability $P^{\beta/h}_{\mathrm{recomb}}(\boldsymbol{E})$ of a recombination event $\boldsymbol{E} = (V, D, J, \mathrm{del}V, \mathrm{del}Dl, \mathrm{del}Dr, \mathrm{del}J, \mathrm{insVD}, \mathrm{insDJ})$ for a TCRβ or BCR heavy chain is:

$$\begin{aligned} P^{\beta/H}_{\mathrm{recomb}}(\boldsymbol{E}) = P(V,D,J)P(\mathrm{del}V|V) \\ \times P(\mathrm{insVD})\mathrm{P}(\mathrm{delDl},\mathrm{delDr}|\mathrm{D}) \\ \times P(\mathrm{insDJ})\mathrm{P}(\mathrm{delJ}|\mathrm{J}) \\ \times \prod_{i}^{\mathrm{insVD}} P_{\mathrm{VD}}(n_i|n_{i-1}) \prod_{i}^{\mathrm{insDJ}} P_{\mathrm{DJ}}(m_i|m_{i-1}). \end{aligned} \tag{2}$$

In the case of TRB, gene usage is further factorized as $P(V, D, J) = P(V)P(D, J)$.

b. General model formulation

IGoR is designed in a modular way so the user can define arbitrary model forms. The models are Bayesian networks encoded as directed acyclic graphs, whose vertices $i = 1, \ldots, K$ label individual recombination subprocesses E_i (V, D, J choices, deletions, etc. in the examples above). Dependence of the recombination process j upon i is encoded by a directed edge between i and j, denoted $v_{ij} = 1$ (while $v_{ij} = 0$ means no direct dependence). The set of parents of i, i.e. processes on which i depends directly, is denoted by $\mathcal{P}_i = \{j|v_{ji} = 1\}$.

Using these definitions we can, generally and irrespectively of the assumed form of the underlying model of recombination, write the probability of a complete recombination scenario $\boldsymbol{E} = (E_1, \ldots, E_K)$ as:

$$P_{\mathrm{recomb}}(\boldsymbol{E}|\theta) = \prod_{i=1}^{K} P(E_i|\{E_j\}_{j\in\mathcal{P}_i}, \theta), \tag{3}$$

where θ denotes the underlying model parameters (i.e. probability distributions of gene choice, insertions at a given junction, and deletions from a given gene in the studied examples).

Each recombination scenario $\boldsymbol{E}$ leads to a unique sequence $\hat{\boldsymbol{S}}(\boldsymbol{E}) = (\hat{S}_1, \ldots, \hat{S}_L)$, $\hat{S}_i(E) \in \{A, C, G, T\}$. However, in order to produce a given sequence $\boldsymbol{S}$ several scenarios might be equiv-

alent, and we can write the probability of generating a given sequence as:

$$P_{\text{gen}}(\boldsymbol{S}|\theta) = \sum_{\boldsymbol{E}|\hat{\boldsymbol{S}}(\boldsymbol{E})=\boldsymbol{S}} P_{\text{recomb}}(\boldsymbol{E}|\theta). \tag{4}$$

The above description only holds to assess the generation probability of a pure product of recombination and does not account for sequencing errors or hypermutations. Note that, since longer reads allow for more reliable determination of V and J gene segments, P_{gen} depends in general on read length: shorter reads can be created in more ways than longer reads, leading to larger P_{gen}.

c. Errors and hypermutations

Sequencing is inherently noisy and introduces nucleotide substitutions. In addition, BCRs can accumulate hypermutations, which can be mathematically treated in the same way as errors. For the sake of clarity, we distinguish between the sequencing read $\boldsymbol{R}$ and the original sequence $\boldsymbol{S}$ resulting from recombination, as defined above. For simplicity we ignore insertion and deletion errors, so that $\boldsymbol{R}$ and $\boldsymbol{S}$ are of the same length L.

We define our error model as deviations from the initial recombination event (through sequencing errors or somatic hypermutations) such that $P_{\text{err}}(\boldsymbol{R}|\boldsymbol{S},\theta)$ is the probability of observing the sequencing read $\boldsymbol{R}$ given the recombination product $\boldsymbol{S}$. Since the recombination scenario $\boldsymbol{E}$ completely determines $\boldsymbol{S}$, $P_{\text{err}}(\boldsymbol{R}|\boldsymbol{S},\theta) = P_{\text{err}}(\boldsymbol{R}|\boldsymbol{E},\theta)$, and we use these two notations interchangeably. The dependence on θ reflects the fact that θ also includes the parameters of the error or hypermutation model.

We write the joint probability of producing a given sequence $\boldsymbol{S}$ and observing a given read $\boldsymbol{R}$ as:

$$P(\boldsymbol{R},\boldsymbol{S}|\theta) = P_{\text{gen}}(\boldsymbol{S}|\theta)P_{\text{err}}(\boldsymbol{R}|\boldsymbol{S},\theta). \tag{5}$$

Summing over all possible recombination products, the likelihood of a sequencing read is:

$$\begin{aligned} P_{\text{read}}(\boldsymbol{R}|\theta) &= \sum_{\boldsymbol{S}} P(\boldsymbol{R},\boldsymbol{S}|\theta) \\ &= \sum_{E} P_{\text{recomb}}(E|\theta)P_{\text{err}}(\boldsymbol{R}|\boldsymbol{E},\theta), \end{aligned} \tag{6}$$

and the total likelihood of the model given a dataset of reads $(\boldsymbol{R}^1,\dots,\boldsymbol{R}^N)$ is given by:

$$\mathcal{L}_{\text{total}}(\theta) = \prod_{a=1}^{N} P_{\text{read}}(\boldsymbol{R}^a|\theta). \tag{7}$$

Supplementary Note 2 – Expectation-maximization

a. General scheme

The recombination machinery is degenerate, as several scenarios of recombination and hypermutations can lead to the same sequence, and the recombination scenario $\boldsymbol{E}$ from which the sequencing read $\boldsymbol{R}$ comes from is in general unknown. The Expectation-Maximization algorithm is a commonly used algorithm that maximizes the likelihood of models with hidden variables given the data. In this section we re-derive this algorithm for our class of models.

The procedure is iterative. Starting from an initial set of parameters θ, one wishes to update another set of parameters θ'. From Bayes formula, $P_{\text{read}}(\boldsymbol{R}|\theta') = P(\boldsymbol{E},\boldsymbol{R}|\theta')/P(\boldsymbol{E}|\boldsymbol{R},\theta')$, we rewrite the log-likelihood of a read as:

$$\ln P_{\text{read}}(\boldsymbol{R}|\theta') = \sum_{\boldsymbol{E}} P(\boldsymbol{E}|\boldsymbol{R},\theta)\left[\ln P(\boldsymbol{E},\boldsymbol{R}|\theta') - \ln P(\boldsymbol{E}|\boldsymbol{R},\theta')\right] = q(\theta'|\theta,\boldsymbol{R}) + h(\theta'|\theta,\boldsymbol{R}), \quad (8)$$

where we have used $\sum_{\boldsymbol{E}} P(\boldsymbol{E}|\boldsymbol{R},\theta) = 1$, and where we have defined

$$h(\theta'|\theta,\boldsymbol{R}) = -\sum_{\boldsymbol{E}} P(\boldsymbol{E}|\boldsymbol{R},\theta)\ln P(\boldsymbol{E}|\boldsymbol{R},\theta'), \quad (9)$$

$$q(\theta'|\theta,\boldsymbol{R}) = \sum_{\boldsymbol{E}} P(\boldsymbol{E}|\boldsymbol{R},\theta)\ln P(\boldsymbol{E},\boldsymbol{R}|\theta'). \quad (10)$$

The difference between the log-likelihood, $\ln \mathcal{L}_{\text{total}}(\theta) = \sum_{a=1}^{N} \ln P_{\text{read}}(\boldsymbol{R}|\theta)$, between the current set of parameters θ and the candidate new parameters θ' reads:

$$\begin{aligned}\ln \mathcal{L}_{\text{total}}(\theta') - \ln \mathcal{L}_{\text{total}}(\theta) &= \sum_{a=1}^{N} q(\theta'|\theta,\boldsymbol{R}^a) - q(\theta|\theta,\boldsymbol{R}^a) + h(\theta'|\theta,\boldsymbol{R}^a) - h(\theta|\theta,\boldsymbol{R}^a).\\ &\geq \sum_{a=1}^{N} q(\theta'|\theta,\boldsymbol{R}^a) - q(\theta|\theta,\boldsymbol{R}^a)\\ &\geq Q(\theta'|\theta) - Q(\theta|\theta)\end{aligned} \quad (11)$$

where $Q(\theta'|\theta) = \sum_{a=1}^{N} q(\theta'|\theta,\boldsymbol{R}^a)$, and where we have used Gibbs inequality:

$$h(\theta'|\theta,\boldsymbol{R}^a) - h(\theta|\theta,\boldsymbol{R}^a) = \sum_{\boldsymbol{E}} P(\boldsymbol{E}|\boldsymbol{R},\theta)\ln\frac{P(\boldsymbol{E}|\boldsymbol{R},\theta)}{P(\boldsymbol{E}|\boldsymbol{R},\theta')} \geq 0. \quad (12)$$

This inequality ensures that maximizing the "pseudo-log-likelihood" $Q(\theta'|\theta)$ over θ' increases total likelihood by at least the same amount. The Expectation-Maximization scheme updates θ by doing such a maximization, and repeating the procedure iteratively. The algorithm converges to a maximum of the likelihood.

b. Optimizing the recombination model

The pseudo-log-likelihood can be broken up in two independent terms, $Q(\theta'|\theta) = Q_{\text{recomb}}(\theta'|\theta) + Q_{\text{err}}(\theta'|\theta)$, respectively corresponding to the recombination model and the error or hypermutation model:

$$Q_{\text{recomb}}(\theta'|\theta) = \sum_{a=1}^{N} \sum_{\boldsymbol{E}} P(\boldsymbol{E}|\boldsymbol{R}^a, \theta) \ln P_{\text{recomb}}(\boldsymbol{E}|\theta'). \tag{13}$$

$$Q_{\text{err}}(\theta'|\theta) = \sum_{a=1}^{N} \sum_{\boldsymbol{E}} P(\boldsymbol{E}|\boldsymbol{R}^a, \theta) \ln P_{\text{err}}(\boldsymbol{R}|\boldsymbol{E}, \theta'). \tag{14}$$

In order to maximize the pseudo-log-likelihood of the recombination model we need to maximize $Q_{\text{recomb}}(\theta'|\theta)$ with respect to every model component contained in the parameter set θ', $P'(E_i|\{E_j\}_{j\in\mathcal{P}_i})$. We impose normalization using Lagrange multipliers, λ_i, and define:

$$\hat{Q}_{\text{recomb}}(\theta'|\theta) = Q_{\text{recomb}}(\theta'|\theta) + \sum_i \lambda_i \left[1 - \sum_{E_i} P'(E_i|\{E_j\}_{j\in\mathcal{P}_i})\right]. \tag{15}$$

Taking the functional derivative of $\hat{Q}_{\text{recomb}}(\theta^*|\theta)$ with respect to the model parameter we get:

$$\frac{\partial \hat{Q}_{\text{recomb}}(\theta'|\theta)}{\partial P'(E_i|\{E_j\}_{j\in\mathcal{P}_i})} = \sum_{a=1}^{N} \sum_{\boldsymbol{E}'} \delta_{E_i,E_i'} \frac{P(\boldsymbol{E}'|\boldsymbol{R}^a, \theta)}{P'(E_i|\{E_j\}_{j\in\mathcal{P}_i})} + \lambda_i. \tag{16}$$

Setting this derivative to zero gives:

$$P'(E_i|\{E_j\}_{j\in\mathcal{P}_i}) = \frac{1}{N} \sum_{a=1}^{N} \sum_{\boldsymbol{E}'} \delta_{E_i,E_i'} P(\boldsymbol{E}'|\boldsymbol{R}^a, \theta), \tag{17}$$

where the Lagrange parameter $\lambda_i = N$ ensures normalization. In other words the modified log-likelihood is maximized by using an update rule that equates the probability of a realization of a recombination event to its posterior frequency.

c. Optimizing the independent single nucleotide error model

The independent single nucleotide error model is the simplest instance of an error model, where each nucleotide of the read has a probability r to be mis-sequenced as one of the three other nucleotides with equal probability. For this model we have

$$P_{\text{err}}(\boldsymbol{R}|\boldsymbol{S}, \theta) = \left(\frac{r}{3}\right)^{N_{\text{err}}} (1 - r)^{L - N_{\text{err}}(\boldsymbol{R},\boldsymbol{S})}. \tag{18}$$

where $N_{\rm err}(\boldsymbol{R},\boldsymbol{S})$ the number of mismatches between $\boldsymbol{R}$ and $\boldsymbol{S}$, and L the number of nucleotides that can be potentially identified by the algorithm as errors. We compute the derivative of the modified log-likelihood of the error model with respect to R^* as:

$$\frac{dQ_{\rm err}(\theta'|\theta)}{dr'} = \sum_{a=1}^{N}\sum_{\boldsymbol{E}} P(\boldsymbol{E}|\boldsymbol{R}^a,\theta)\left(\frac{N_{\rm err}(\boldsymbol{R}^a,\hat{\boldsymbol{S}}(\boldsymbol{E}))}{r'} - \frac{L(\boldsymbol{R}^a,\boldsymbol{E}) - N_{\rm err}(\boldsymbol{R}^a,\hat{\boldsymbol{S}}(\boldsymbol{E}))}{1-r'}\right). \quad (19)$$

Setting this derivative to zero yields:

$$R' = \frac{\sum_{a=1}^{N}\sum_{\boldsymbol{E}} P(\boldsymbol{E}|\boldsymbol{R}^a,\theta) N_{\rm err}(\boldsymbol{R}^a,\hat{\boldsymbol{S}}(\boldsymbol{E}))}{\sum_{a=1}^{N}\sum_{\boldsymbol{E}} P(\boldsymbol{E}|\boldsymbol{R}^a,\theta) L(\boldsymbol{R}^a,\boldsymbol{E})}, \quad (20)$$

where $L(\boldsymbol{R}^a,\boldsymbol{E})$ is the number of potentially erroneous nucleotides in read a. For simplicity we ignore errors and hypermutations in the insertion part of the sequence, as they are almost indistinguishable from unmutated random insertions, and accounting for them would imply summing over an exponentially large number of scenarios. As a result, L in the above formula is not the read length, but rather the number of germline nucleotides in each scenario, which depends on the scenario $\boldsymbol{E}$ as well as on the sequence read.

d. Optimizing the hypermutation model

The hypermutation model assumes the following form for the probability of hypermutations:

$$P_{\rm err}(\boldsymbol{R}|\boldsymbol{S}) = \prod_{x,S_x\neq R_x} \frac{P_{\rm mut}(S_{x-m},\dots,S_{x+m})}{3} \prod_{x,S_x=R_x} \left[1 - P_{\rm mut}(S_{x-m},\dots,S_{x+m})\right], \quad (21)$$

with

$$\frac{P_{\rm mut}(\boldsymbol{\pi})}{1-P_{\rm mut}(\boldsymbol{\pi})} = \mu \exp\left(\sum_{i=-m}^{m} e_i(\pi_i)\right), \quad (22)$$

where $(\pi_{-m},\dots,\pi_m) = (S_{x-m},\dots,S_{x+m})$ is the sequence context of the original recombination product around a hypermutation at position x. The parameters $e_i(N)$, the position-weight matrix, and μ, the overall mutation rate, are part of the parameter set θ. In order to lift the degeneracy of the model we impose that $\sum_{N=A,C,G,T} e_i(N) = 0$ at every position i.

The pseudo-log-likelihood of the hypermutation model reads:

$$Q_{\rm err}(\theta'|\theta) = \sum_{a=1}^{M}\sum_{\boldsymbol{E}} P(\boldsymbol{E}|\boldsymbol{R}^a,\theta)\sum_{x=1}^{L}\left[\delta_{S_x,R_x}\ln\frac{1}{1+r'(\hat{\boldsymbol{S}}(\boldsymbol{E}),x)} + (1-\delta_{S_x,R_x})\ln\frac{r'(\hat{\boldsymbol{S}}(\boldsymbol{E}),x)/3}{(1+r'(\hat{\boldsymbol{S}}(\boldsymbol{E}),x))}\right], \quad (23)$$

where $r'(S,x) = r'(S_{x-m},\ldots,S_{x+m}) = \mu' \exp\left(\sum_{i=-m}^{m} e'_i(S_{x+i})\right)$. It can be rewritten as:

$$Q_{\text{err}}(\theta'|\theta) = \sum_{\boldsymbol{\pi}} \left[\left(\ln(\mu'/3) + \sum_{i=0}^{N} e'_i(\pi_i) \right) N_{\text{mut}}(\boldsymbol{\pi}) - \ln\left(1 + \mu' \exp\left(\sum_{i=1}^{N} e'(\pi_i)\right)\right) N_{\text{bg}}(\boldsymbol{\pi}) \right], \tag{24}$$

where

$$N_{\text{bg}}(\boldsymbol{\pi}) = \sum_{a=1}^{M} \sum_{\boldsymbol{E}} P(\boldsymbol{E}|\boldsymbol{R}^a,\theta) \sum_{x=1}^{L} \prod_{i=-m}^{m} \delta_{\hat{S}_{x+i}(\boldsymbol{E}),\pi_i} \tag{25}$$

$$N_{\text{mut}}(\boldsymbol{\pi}) = \sum_{a=1}^{M} \sum_{\boldsymbol{E}} P(\boldsymbol{E}|\boldsymbol{R}^a,\theta) \sum_{x=1}^{L} (1 - \delta_{\hat{S}_x(\boldsymbol{E}),R_x}) \prod_{i=-m}^{m} \delta_{\hat{S}_{x+i}(\boldsymbol{E}),\pi_i}. \tag{26}$$

During the Expectation step, we compute these two quantities for each (2m+1)-mer and then maximize Q_{err} at each step of the Expectation-Maximization scheme using Newton's method with a backtracking line search. To impose $\sum_\sigma e_i(\sigma) = 0$ we remove one parameter per position i by setting for one nucleotide, $e_i(N) = -\sum_{\sigma\neq N} e_i(\sigma)$.

We can then compute the entries of the gradient vector $\boldsymbol{J}$ (of size $3(2m+1)+1$):

$$\frac{\partial Q_{\text{err}}(\theta'|\theta)}{\partial \mu'} = \sum_{\boldsymbol{\pi}} \left(\frac{N_{\text{mut}}(\boldsymbol{\pi})}{\mu'} - N_{\text{bg}}(\boldsymbol{\pi}) \frac{r'(\boldsymbol{\pi})}{\mu'(1+r'(\boldsymbol{\pi}))} \right), \tag{27}$$

$$\frac{\partial Q_{\text{err}}(\theta'|\theta)}{\partial e'_i(\sigma)} = \sum_{\boldsymbol{\pi}} (\delta_{\boldsymbol{\pi}_i,\sigma} - \delta_{\boldsymbol{\pi}_i,N}) \left[N_{\text{mut}}(\boldsymbol{\pi}) - N_{\text{bg}}(\boldsymbol{\pi}) \frac{r'(\boldsymbol{\pi})}{1+r'(\boldsymbol{\pi})} \right], \tag{28}$$

along with the Hessian matrix $\boldsymbol{H}$ entries:

$$\frac{\partial^2 Q_{\text{err}}(\theta'|\theta)}{\partial \mu'^2} = \sum_{\boldsymbol{\pi}} \left(N_{\text{bg}}(\boldsymbol{\pi}) \frac{r'(\boldsymbol{\pi})^2}{\mu'^2(1+r'(\boldsymbol{\pi}))^2} - \frac{N_{\text{mut}}(\boldsymbol{\pi})}{\mu'^2} \right), \tag{29}$$

$$\frac{\partial^2 Q_{\text{err}}(\theta'|\theta)}{\partial \mu' \partial e'_i(\sigma)} = \sum_{\boldsymbol{\pi}} (\delta_{\boldsymbol{\pi}_i,N} - \delta_{\boldsymbol{\pi}_i,\sigma}) N_{\text{bg}}(\boldsymbol{\pi}) \frac{r'(\boldsymbol{\pi})}{\mu'(1+r'(\boldsymbol{\pi}))^2}, \tag{30}$$

$$\frac{\partial^2 Q_{\text{err}}(\theta'|\theta)}{\partial e'_i(\sigma) \partial e'_j(\sigma')} = \sum_{\boldsymbol{\pi}} (\delta_{\boldsymbol{\pi}_i,N} - \delta_{\boldsymbol{\pi}_i,\sigma})(\delta_{\boldsymbol{\pi}_j,N} - \delta_{\boldsymbol{\pi}_j,\sigma'}) N_{\text{bg}}(\boldsymbol{\pi}) \frac{r'(\boldsymbol{\pi})}{(1+r'(\boldsymbol{\pi}))^2}. \tag{31}$$

For each step of Newton's method we find the step direction by solving $\boldsymbol{H}\Delta\theta' = -\boldsymbol{J}$ and we gradually refine the step size based on the Armijo-Goldstein condition. These operations are iteratively repeated until the pseudo-log-likelihood of the error model for a given Maximization step of the EM framework is maximized.

We also inferred a non-additive hypermutation model, parametrized by the full function $P_{\text{mut}}(\boldsymbol{\pi})$ of the 5-mer context $\boldsymbol{\pi}$. The EM iteration step then takes a much simpler form:

$$P_{\text{mut}}(\boldsymbol{\pi}) \leftarrow \frac{N_{\text{mut}}(\boldsymbol{\pi})}{N_{\text{bg}}(\boldsymbol{\pi})}. \tag{32}$$

where $N_{\text{bg}}(\boldsymbol{\pi})$ and $N_{\text{mut}}(\boldsymbol{\pi})$ are given by Eqs. 25 and 26.

Supplementary Note 3 – Model entropy and D_{KL}

Shannon's entropy [4, 5],

$$S(\theta) = \sum_x p(x|\theta) \ln p(x|\theta), \tag{33}$$

is a measure of the uncertainty about the outcome of a stochastic process described by a variable x, governed by the distribution $p(x|\theta)$ and parametrized by θ. As in [1, 2, 6] we compute this quantity based on our probabilistic framework and use it as an estimate for the diversity generated by the V(D)J recombination process. In the main text we also introduced the relative entropy or Kullback-Leibler divergence,

$$D(\theta_1||\theta_2) = \sum_x p(x|\theta_1) \ln \frac{p(x|\theta_1)}{p(x|\theta_2)}, \tag{34}$$

as a measure of dissimilarity between two probability distributions parametrized by θ_1 and θ_2 respectively, and used it to quantify the error made by our probabilistic framework upon inferring the V(D)J recombination parameters.

Since both the entropy and the Kullback Leibler divergence between two recombination models can be computed once one knows how to compute the cross entropy $H(\theta_1, \theta_2) = \sum_x p(x|\theta_1) \ln p(x|\theta_2)$ between the distributions for the two sets of parameters θ_1 and θ_2, we focus here on the computation of $H(\theta_1, \theta_2)$.

a. General form

For the considered class of models, the cross-entropy can be divided into subparts for each model component,

$$H(\theta_1, \theta_2) = \sum_{i=1}^{K} H_i(\theta_1, \theta_2), \tag{35}$$

with

$$H_i(\theta_1, \theta_2) = \sum_{\boldsymbol{E}} P(\boldsymbol{E}|\theta_1) \ln P(E_i|\{E_j\}_{j\in\mathcal{P}_i}, \theta_2). \tag{36}$$

To calculate this sum, one does not need to sum over all possible scenarios $\boldsymbol{E}$, but only on combinations of processes that affect E_i directly or indirectly. Let us call $A_i \subset \{1, \ldots, K\}$ the set of indices affecting process i. These are defined as the "ancestors" of i in the acyclic graph, i.e. indices j such that there exists a lineage from j to i, $(i_1 = i, i_2, \ldots, i_k = j)$ with $i_{\ell+1} \in \mathcal{P}_{i_\ell}$ (note

that A_i includes i itself as a 0th order ancestor). Then the previous sum can be reduced to a sum over the processes in A only:

$$H_i(\theta_1,\theta_2) = \sum_{\boldsymbol{E}_{A_i}} \left[\prod_{j\in A_i} P(E_j|\{E_{j'}\}_{j'\in\mathcal{P}_j},\theta_1) \right] \ln P(E_i|\{E_j\}_{j\in\mathcal{P}_i},\theta_2). \tag{37}$$

where $\boldsymbol{E}_{A_i}$ denotes the subvector of elements of $\boldsymbol{E}$ with indices in A. Estimating the cross entropy for an event E_i requires exponential time in the number of ancestors of that node. Fortunately, in the recombination models considered in this paper the set of ancestors are small and obtaining the cross entropy is easy for every event. The special case of insertions is discussed below. Note that this cross-entropy only takes into account the recombination model, and not the error model.

b. Inserted nucleotides

For a given insertion length insVJ (or insVD, or insDJ), the cross-entropy between two models of insertions is given by

$$h(\text{insVJ},\theta_1,\theta_2) = \sum_{\boldsymbol{n}} P(\boldsymbol{n},\theta_1) \ln P(\boldsymbol{n},\theta_2) \tag{38}$$

$$= \sum_{n_1} P_s(n_1|\theta_1) \ln P_s(n_1|\theta_2) \tag{39}$$

$$+ (\text{insVJ}-1) \sum_{n_1,n_2} P_s(n_1|\theta_1) P(n_2|n_1,\theta_1) \ln P(n_2|n_1,\theta_2) \tag{40}$$

where $\boldsymbol{n} = (n_1,\ldots,n_{\text{insVJ}})$ is the inserted sequence, and $P_s(n_1,\theta)$ is the stationary distribution of the Markov chain of insertions, solution of the equation $\sum_{n_0} P(n_1|n_0,\theta) P_s(n_0,\theta) = P_s(n_1,\theta)$. The average cross-entropy over possible lengths is then given by:

$$H_{\text{VJ insertions}}(\theta_1,\theta_2) = \sum_{\boldsymbol{E}_B} \left[\prod_{j\in B} P(E_j|\{E_{j'}\}_{j'\in\mathcal{P}_i},\theta_1) \right] h(\text{insVJ},\theta_1,\theta_2), \tag{41}$$

where $B \subset \{1,\ldots,K\}$ is the subset of processes affecting either insVJ or $\boldsymbol{n}$, exluding insVJ itself.

Supplementary Note 4 – Probability of generation

Although the probability of generation of a sequence without errors or hypermutations is well defined, computing the probability of generation of a mutated sequence, before mutations occurred, is strictly speaking not possible because that sequence is not known with certainty. How-

ever, we can compute a good approximation for it, and we can also calculate its distribution across sequences.

To approximate $P_{\rm gen}(\boldsymbol{S})$ from a noisy or hypermutated sequence $\boldsymbol{R}$, we take its geometric average weighted by the probability of the recombination product $\boldsymbol{S}$:

$$\ln P^*_{\rm gen}(\boldsymbol{R}) \approx \sum_{\boldsymbol{E}} P(\boldsymbol{E}|\boldsymbol{R},\theta) \ln P_{\rm gen}(\hat{\boldsymbol{S}}(\boldsymbol{E}),\theta), \tag{42}$$

with $P(\boldsymbol{E}|\boldsymbol{R},\theta) = P_{\rm recomb}(\boldsymbol{E},\theta)P_{\rm err}(\boldsymbol{R}|\hat{\boldsymbol{S}}(\boldsymbol{E}),\theta)/P_{\rm read}(\boldsymbol{R},\theta)$. Alternatively, one can take the generation probability of the most likely recombination product:

$$P^*_{\rm gen}(\boldsymbol{R}) \approx P_{\rm gen}(\boldsymbol{S}^*,\theta), \tag{43}$$

where $\boldsymbol{S}^* = \mathrm{argmax}_{\boldsymbol{S}}\, P(\boldsymbol{S}|\boldsymbol{R},\theta)$.

The distribution $\rho(x)$ of the log-probabilities of generation, $x = \log P_{\rm gen}$, can be computed from data using:

$$\rho(x) = \frac{1}{N}\sum_{a=1}^{N}\sum_{\boldsymbol{E}} P(\boldsymbol{E}|\boldsymbol{R},\theta)\delta\left[x - \ln P_{\rm gen}(\hat{\boldsymbol{S}}(\boldsymbol{E}),\theta)\right]. \tag{44}$$

Note that unlike estimates for single sequences, this expression should become exact in the limit of $N \to \infty$.

Supplementary Note 5 – Data and software

a. Germline templates

We used custom germline templates derived from the IMGT database [7]. TCR alpha V and J germline templates were taken from the IMGT human database. For TCR beta V, D and J genes we used curated germline templates from [1]. BCR heavy chain V, D and J genes were taken from the customized germline templates used in [2]. For software comparison we used the same germline templates as in IGoR.

b. Alignments

Initial alignments to germline genes were performed using the Smith-Waterman algorithm [8], with scores of 5 for matching base pairs, -14 for mismatches, and a 50 gap penalty. Alignments

with a score below the following gene dependent threshold were discarded: 50 for TRBV, 0 for TRBD, 10 for TRBJ, 20 for TRAV, 10 for TRAJ, 50 for IGHV, 40 for IGHD, 10 for IGHJ. We also discarded alignments whose score fell below the maximum alignment score (found for this read and segment type), minus the following variable range: 55 for TRBV, 35 for TRBD, 10 for TRBJ, 55 for IGHV, 20 for IGHJ.

The alignment offset (the index of the nucleotide on the read to which the first letter of the undeleted germline template is aligned) was constrained depending on known primer locations on the J gene.

c. *Pruning the tree of scenarios*

Since enumerating all possible scenarios for each sequence is not tractable, we used a heuristic method for reducing their numbers. Exploring all possible scenarios is equivalent to exploring all the terminal leaves of a tree. Our heuristic is to prune all branches that do not contribute substantially to the likelihood of the read. To do this we implement a Sparse Expectation Maximization algorithm as motivated in [9]. Due to the acyclicity of the directed graph underlining the Bayesian network, there exists a topological sorting of the events constituting a partially ordered set (we will assume in the following that the indices of the different events E_i respect this ordering). IGoR processes event realizations according to this order corresponding to different layers of depth in the tree. To discard irrelevant branches (containing negligible scenarios) IGoR computes at each depth k (with $0 \leq k < K$) an upper bound on the probability of the currently explored scenario:

$$\frac{\prod_{0 \leq i \leq k} P(E_i, \boldsymbol{R} | \{E_j\}_{j \in \mathcal{P}_i}, \theta) \prod_{k < i < K} \max_{e_i} P(E_i, \boldsymbol{R} | \theta)}{\max_{\boldsymbol{E} \in \mathcal{E}} P(\boldsymbol{E}, \boldsymbol{R} | \theta)} > \varepsilon, \tag{45}$$

where $\mathcal{E}$ is the set of already fully explored scenarios, and $0 \leq \varepsilon \leq 1$ is a tunable parameter setting the precision of the sparsity approximation. While $\varepsilon = 0$ will explore every possible scenario and perform an exact Expectation step, $\varepsilon = 1$ will explore only scenarios more likely that any scenario already explored.

Although Eq. 45 captures the essence behind our tree pruning approach, in practice IGoR uses more information than a simple upper probability bound. By picking two gene choice realizations, imposing the identity and position of these specific V and J genes, we explicitly impose the total nucleotide length of event realizations between those V and J genes (number of insertions,

deletions, D gene length, ...). When computing the probability upper-bounds IGoR computes the upper probability bound for a given junction length between two event realizations, and uses this refined bound to efficiently prune the tree of scenarios.

d. Generating synthetic sequences

Synthetic sequences are generated by randomly drawing scenarios of recombination from the probability distribution in Eq. 1 or 2. In order to fit the data, the resulting sequences are then cut to mimic the sequencing process (e.g. fixed starting point and fixed read length).

e. Comparison to other software

We benchmarked our method against MiXCR 2.0.2 [10] – a commonly used deterministic alignment method. We used the MiXCR sequence assignment to compute the frequency of gene usage, insertion length, deletions and obtain the distributions shown in Supplementary Fig. 13. We also compared to Partis [11] – a recent HMM based model of recombination. Since Partis uses a Viterbi learning algorithm, we used the most likely assignments it outputs to compute the corresponding probability distribution shown in Supplementary Fig. 13. Since Partis is designed to handle BCRs we assessed its performance on the BCR dataset only.

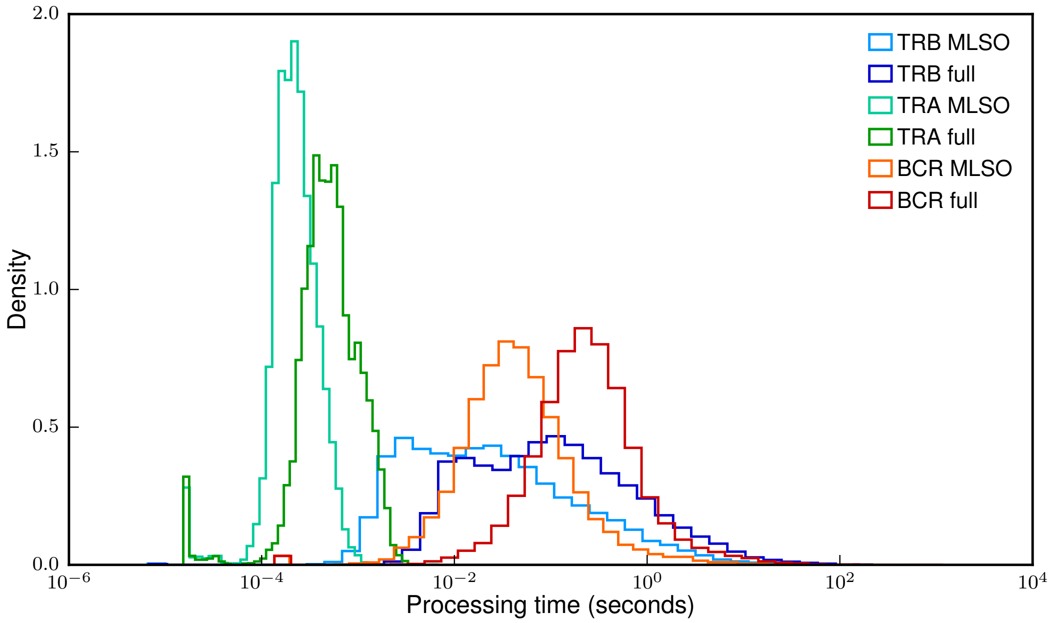

Supplementary Figure 1: **Distribution of the processing time per sequence.** Distribution of the processing time for finding the Most Likely Scenario Only (MLSO) and to evaluate all scenarios (full) for the different chains. Histograms were computed on 20000 sequences for each chain on a single core of an Intel(R) Xeon(R) CPU E5-2680 v3 2.50GHz processor running code compiled with gcc (Debian 4.9.2-10). We benchmarked IGoR's performance for evaluating possible recombination scenarios on real data sequences used to infer the models presented in the main text. We used 60bp TRB sequences for benchmarking since the difficulty for finding the correct V and J for alignment is higher. Finding the Most Likely Scenario Only (MLSO) is on average 3$\times$ faster than evaluating all possible scenarios. Restricting possible scenarios to deterministically assigned V and J genes is on average 6$\times$ faster (data not shown).

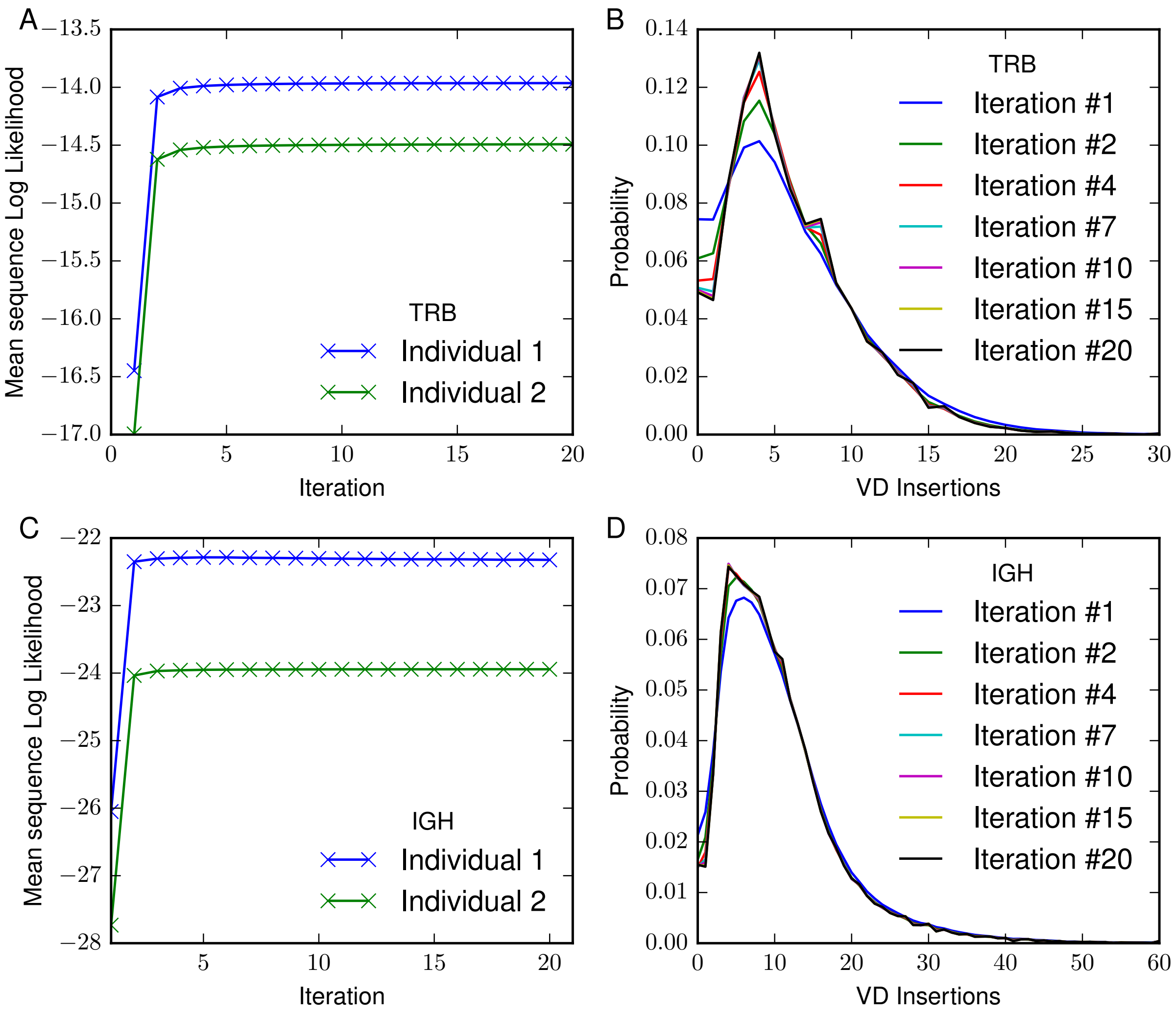

Supplementary Figure 2: **Convergence of IGoR for TRB and naive IGH datasets.** **A.** The mean log likelihood per sequence increases and quickly plateaus, thus reaching the maximum likelihood estimate of the parameters for TRB . **B.** Convergence of the distribution is shown with the example of the distribution of number of VD insertions for TRB. **C.** and **D.** The same figures as **A.** and **B.** for IGH.

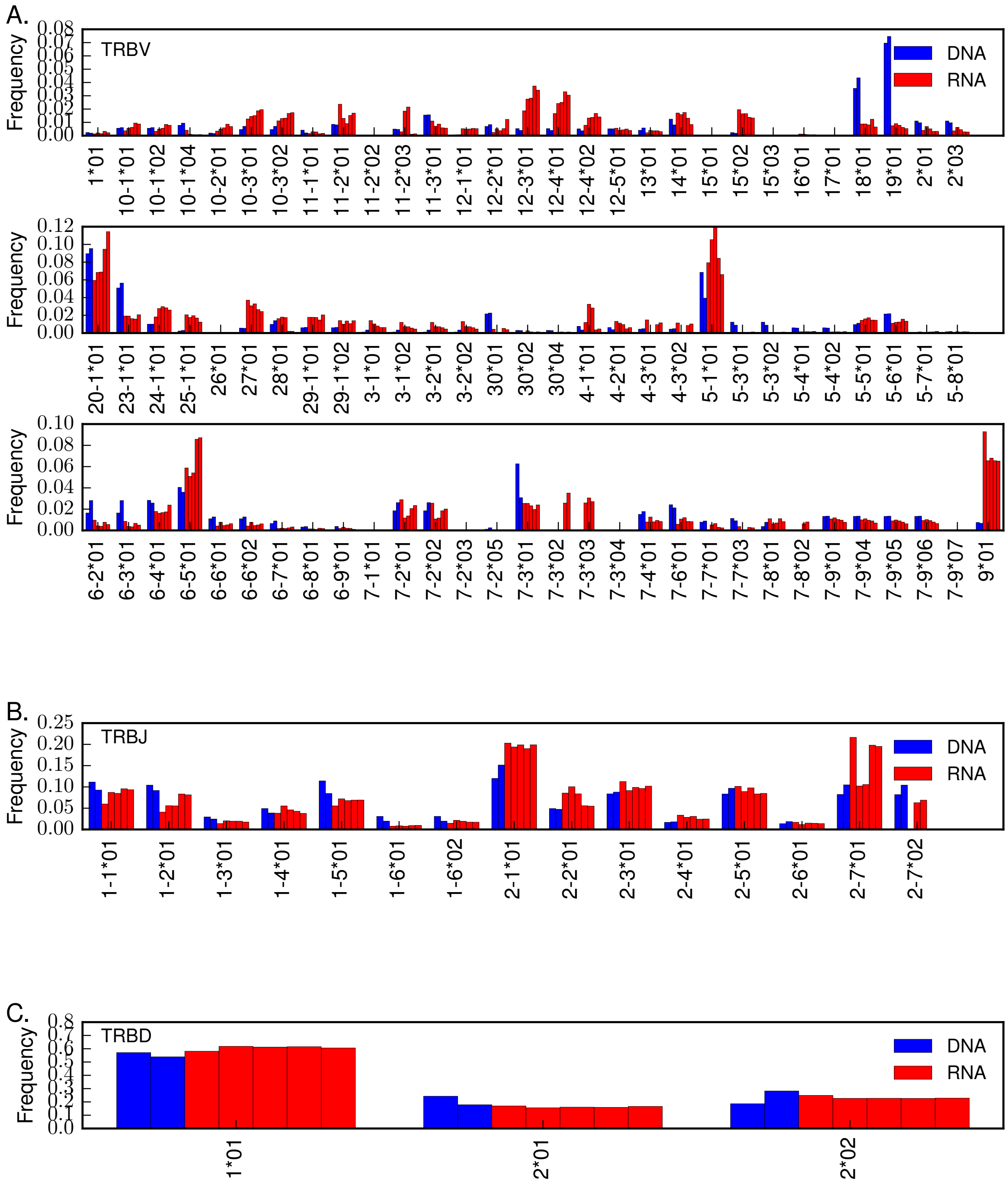

Supplementary Figure 3: **Gene usage in TRB mRNA vs DNA data.** We plot the marginal gene usage averaged over conditional dependencies for V, D and J genes respectively inferred using IGoR from mRNA 100bp (red, [3]) and DNA 60bp (blue, [12]) technology datasets. We observe a higher inter-method than inter-individual variability.

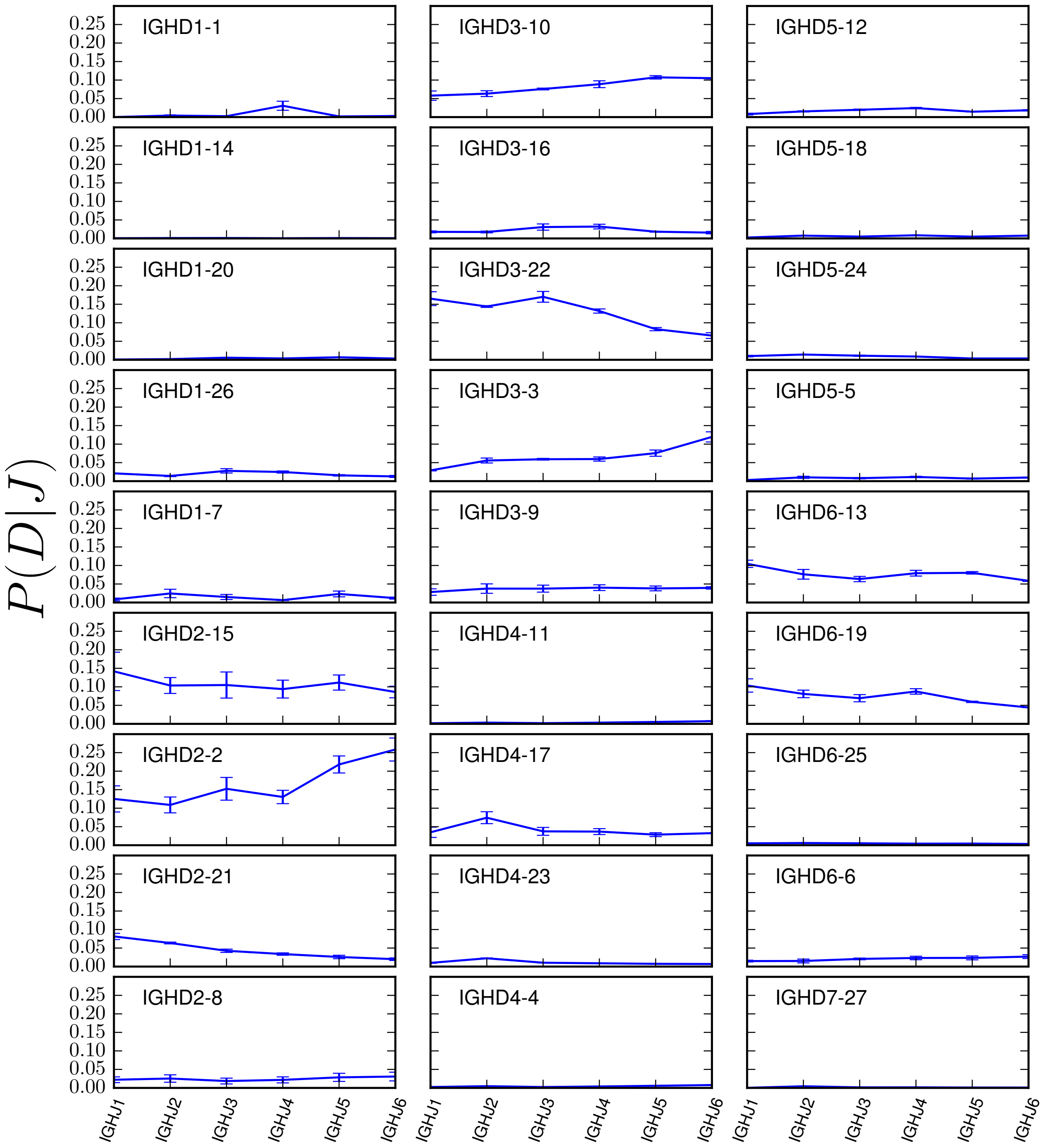

Supplementary Figure 4: **IGH D-J association.** Conditional probability $P(D|J)$ of each D segment, conditioned on the choice of the possible J segments (x axis) in IGH. This figure is the IGH equivalent of Fig. 4d of the main text, which was for TRB. IGH does not exhibit such a clear exlusion rules as TRB, although a dependency exists.

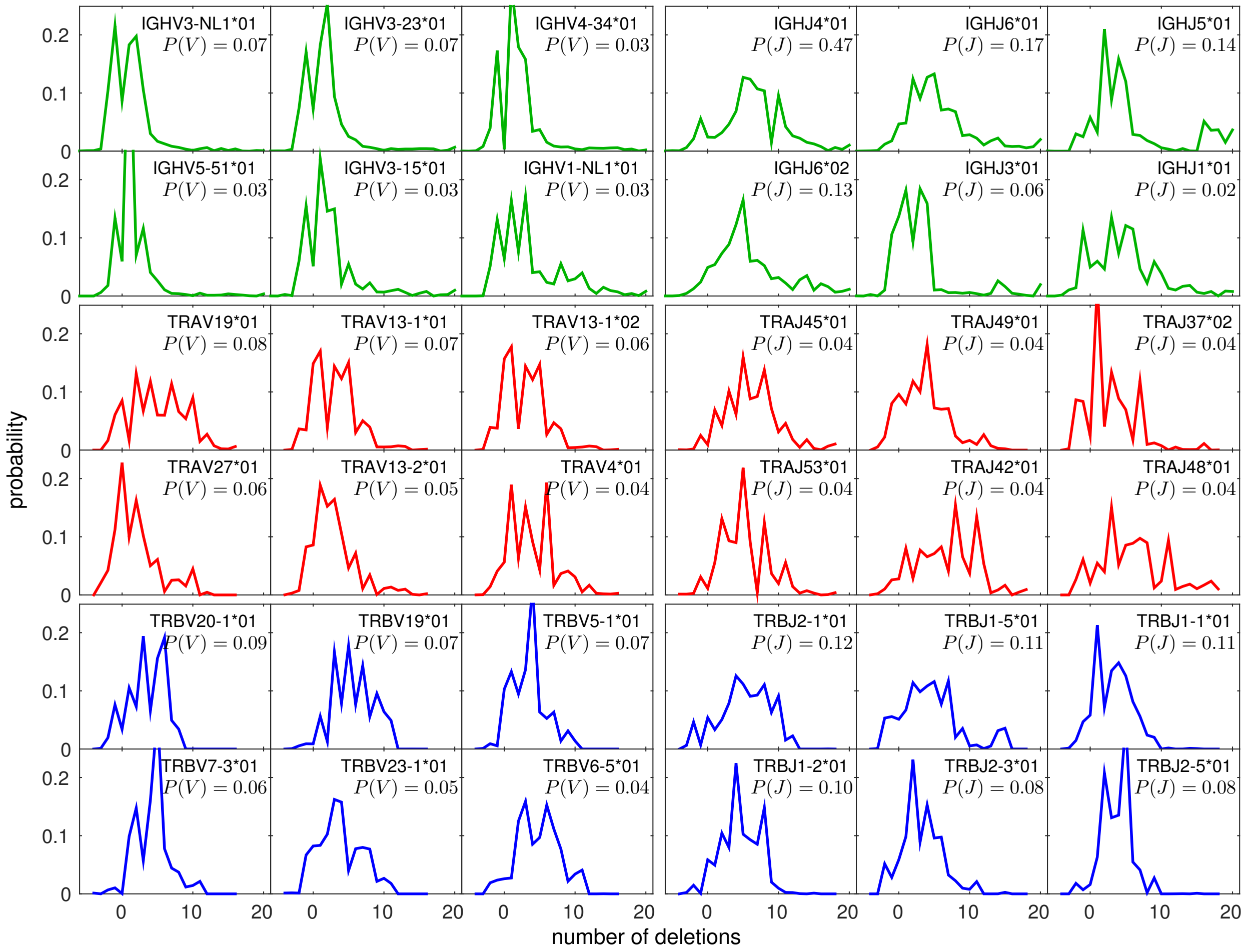

Supplementary Figure 5: **Gene-specific deletion profiles.** Distributions of the number of deletions for each of the 6 most abundant V and J gene segments for the three chains considered: IGH (green), TRA (red), and TRB (blue). Negative deletions represent the number of palindromic insertions. Gene names and their usage frequency are reported within the figures.

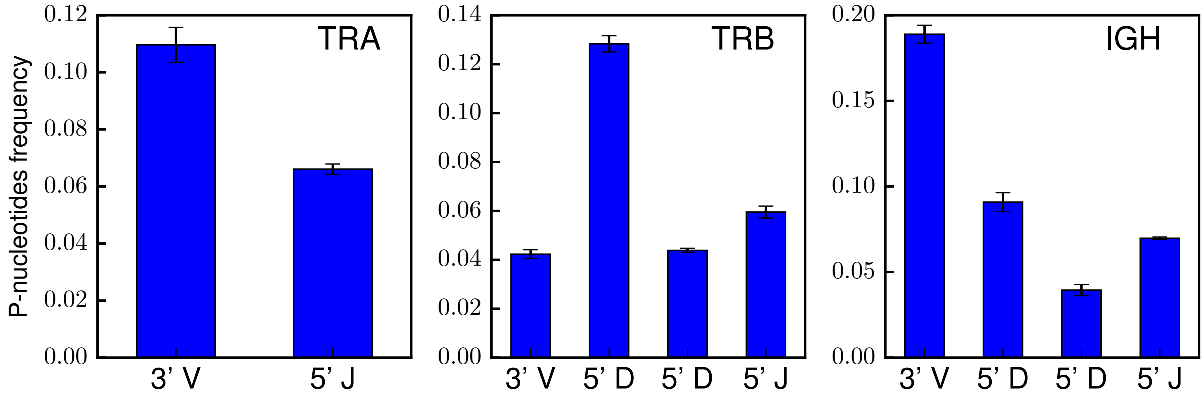

Supplementary Figure 6: **Fraction of germline segment ends with palindromic insertions.** Palindromic insertions are encoded as negative deletions in our framework. For each gene segment and each end (5' or 3'), the bar shows the probability that the end had palindromic insertions, for the TRA, TRB, and IGH chains.

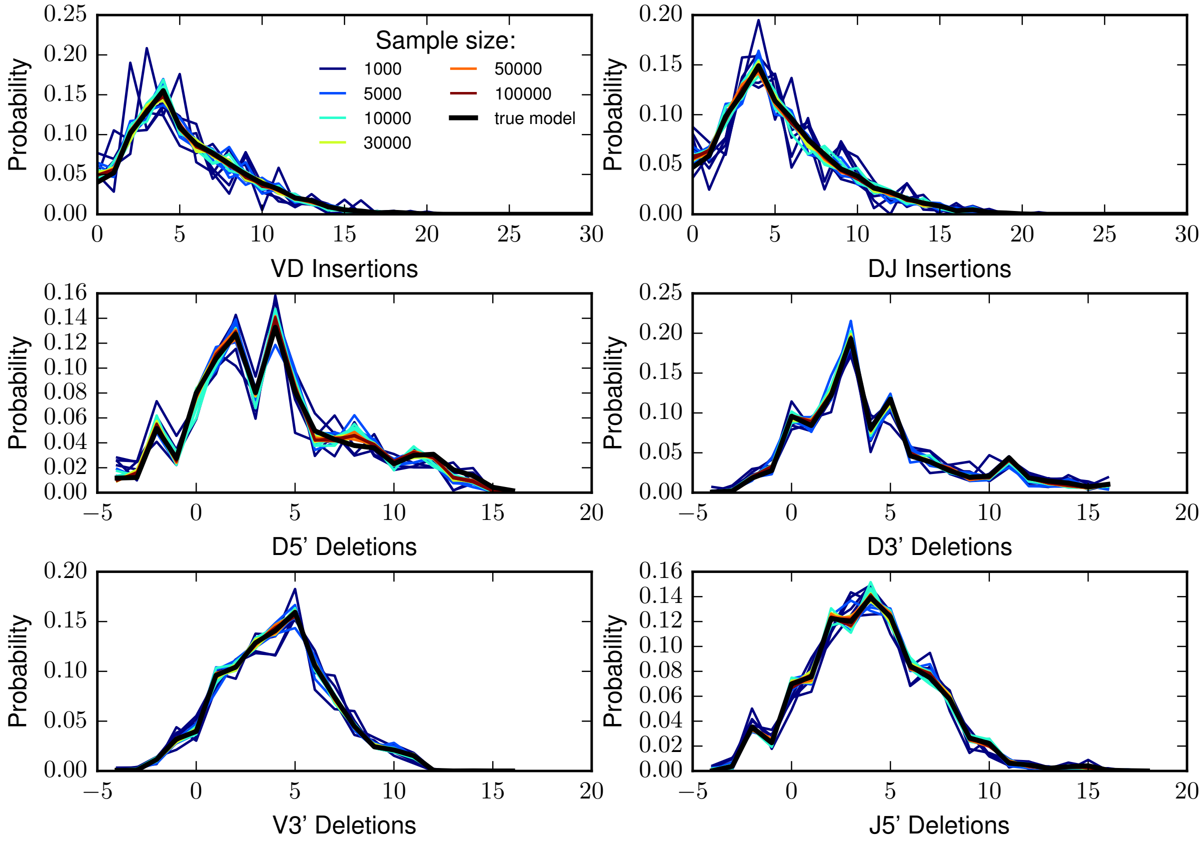

Supplementary Figure 7: **IGoR convergence to the true distribution of TRB recombination for various sample sizes.** Insertion and deletion distributions obtained from 60bp TRB generated samples of various sizes, compared to the true model.

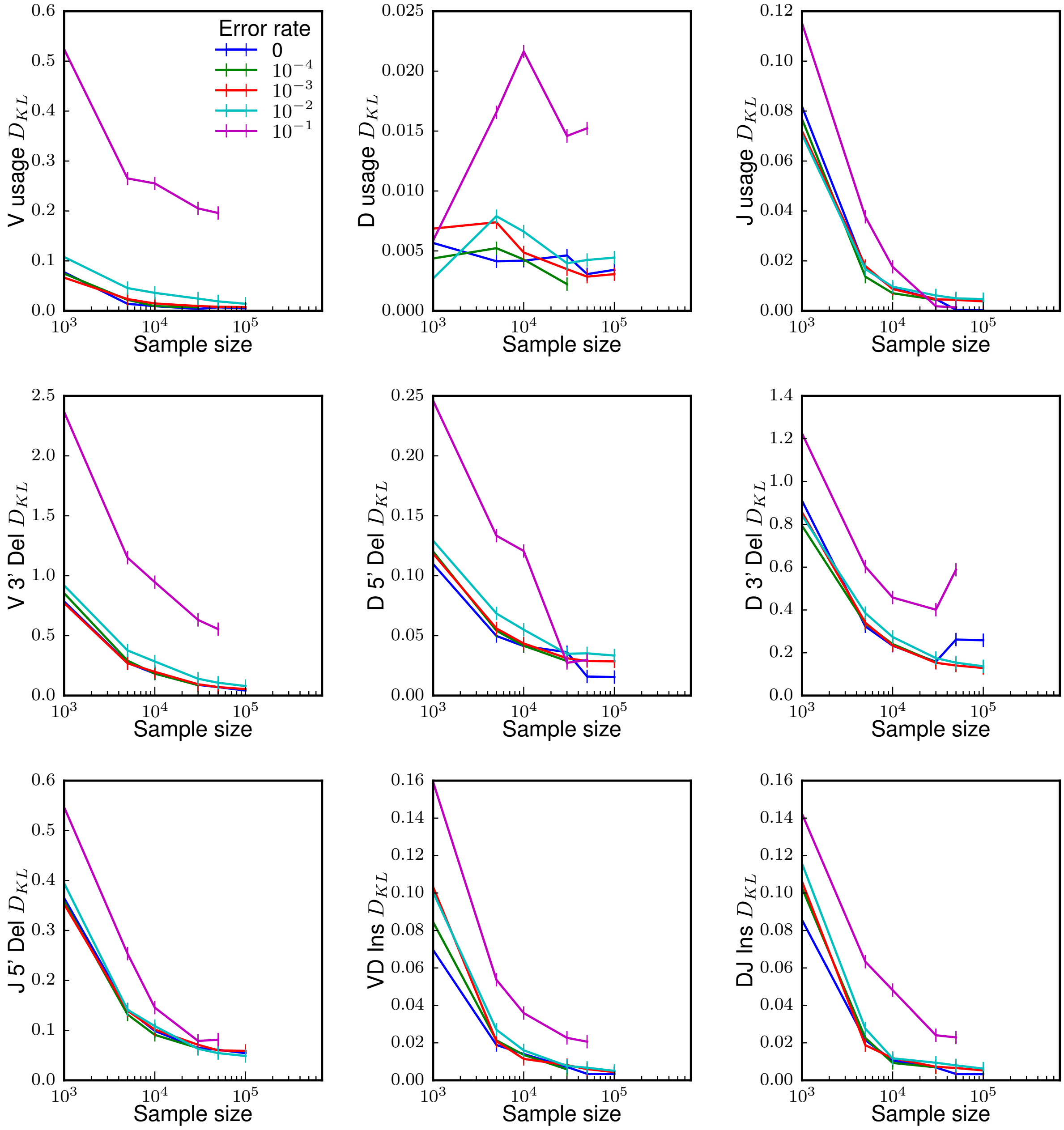

Supplementary Figure 8: **Inference accuracy increases with sample size for TRB.** Kullback-Leibler divergence ($D_{\mathrm{KL}}(\text{inferred} \mid\mid \text{true})$, in bits) between the data-inferred model distributions and the true model distributions, for various features of recombination. Models were inferred using different numbers of sequences and error rates. All components reach a small divergence for sufficiently large sample sizes.

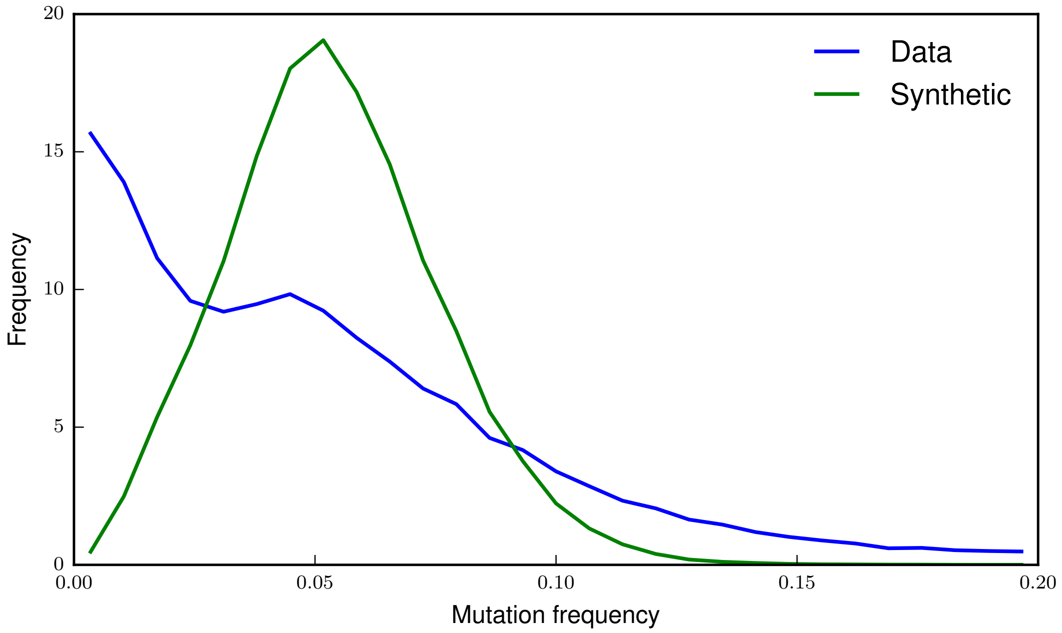

Supplementary Figure 9: **Distribution of hypermutation rates.** Distribution across sequences of the sequence-specific frequency of hypermutations – estimated as the number of hypermutations called by IGoR divided by the total number of germline nucleotides called by IGoR, averaged over possible scenarios for each sequence.

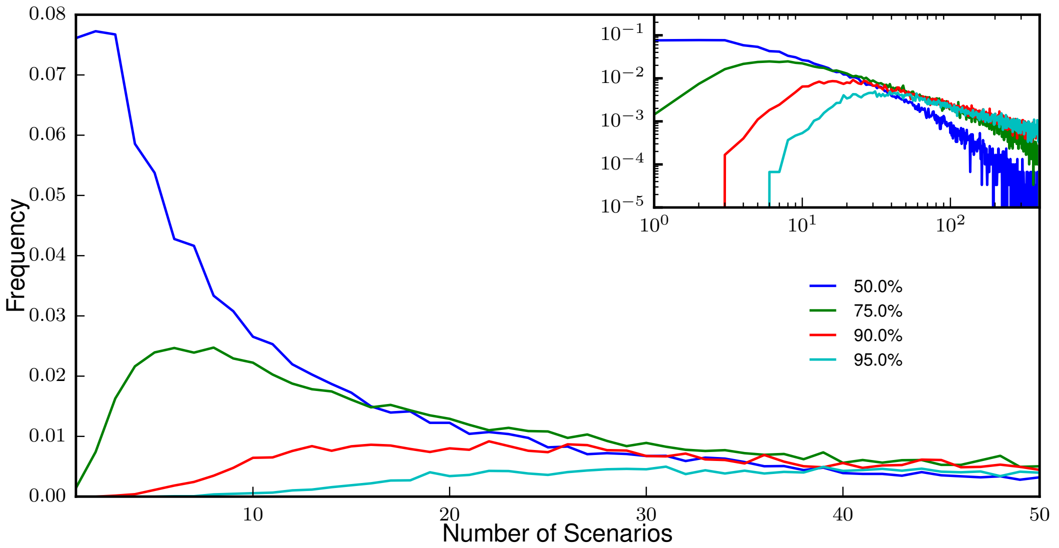

Supplementary Figure 10: **A probabilistic assignment approach is crucial for TCRs.** Equivalent of main text Fig. 4b for 30000 60bp TRB sequences. Distribution of the number of scenarios that need to be enumerated (from most to least likely) to include the true scenario with 50% (blue), 75% (green), 90% (red), or 95% (cyan) confidence. The shorter read length compared to 130bp BCRs entail a higher uncertainty on the V gene identity, for which a higher number of scenarios must be considered.

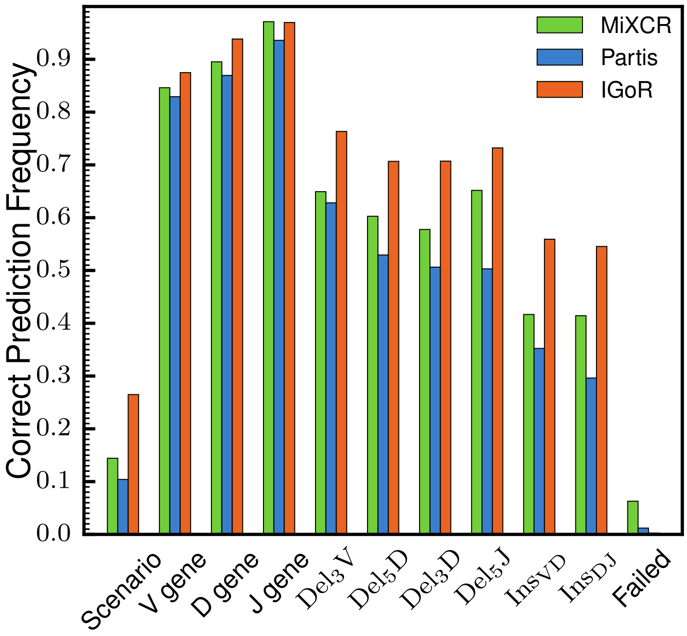

Supplementary Figure 11: **Assignment performance on hypermutation-free IGH sequences without palindromic insertions.** We have shown in main text Fig. 4c the performance of MiXCR, Partis and IGoR at predicting the correct scenario of recombination. Since Partis does not model palindromic insertions, here we show the performance of the three software on sequences that were generated without any palindromic insertions, allowing Partis' performance to be comparable to that of MiXCR.

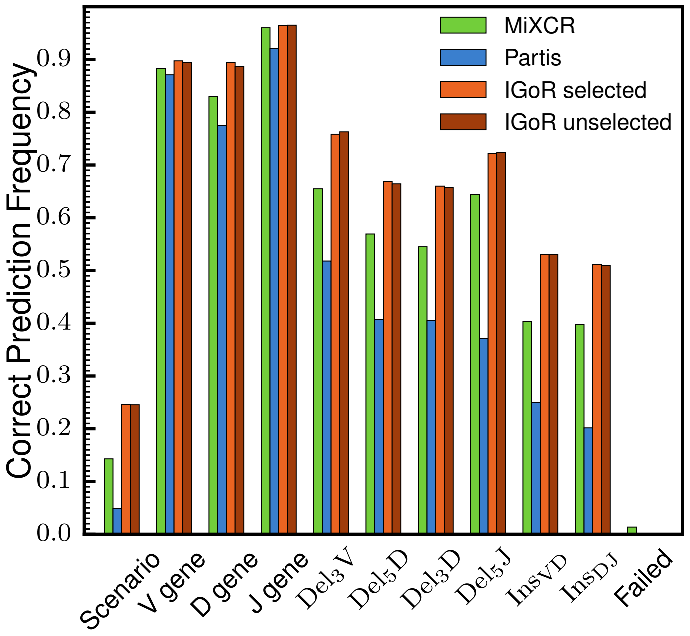

Supplementary Figure 12: **Assignment performance on selected sequences.** IGH sequences were generated by IGoR and artificially selected according to their CDR3 length, so that the resulting distribution of length exactly matches that of the naive in-frame sequences. These sequences were then annotated using IGoR, Partis and MiXCR. For IGoR, scenario assignment was done using two distinct probabilistic models of generation: one that was inferred from unselected sequences (dark red), and one that was learned from selected sequences (light red). Partis used parameters learned from the selected sequences. MiXCR does not need to learn parameters from the data, and was run using the same parameters as for unselected sequences (Fig. 4C of the main text.)

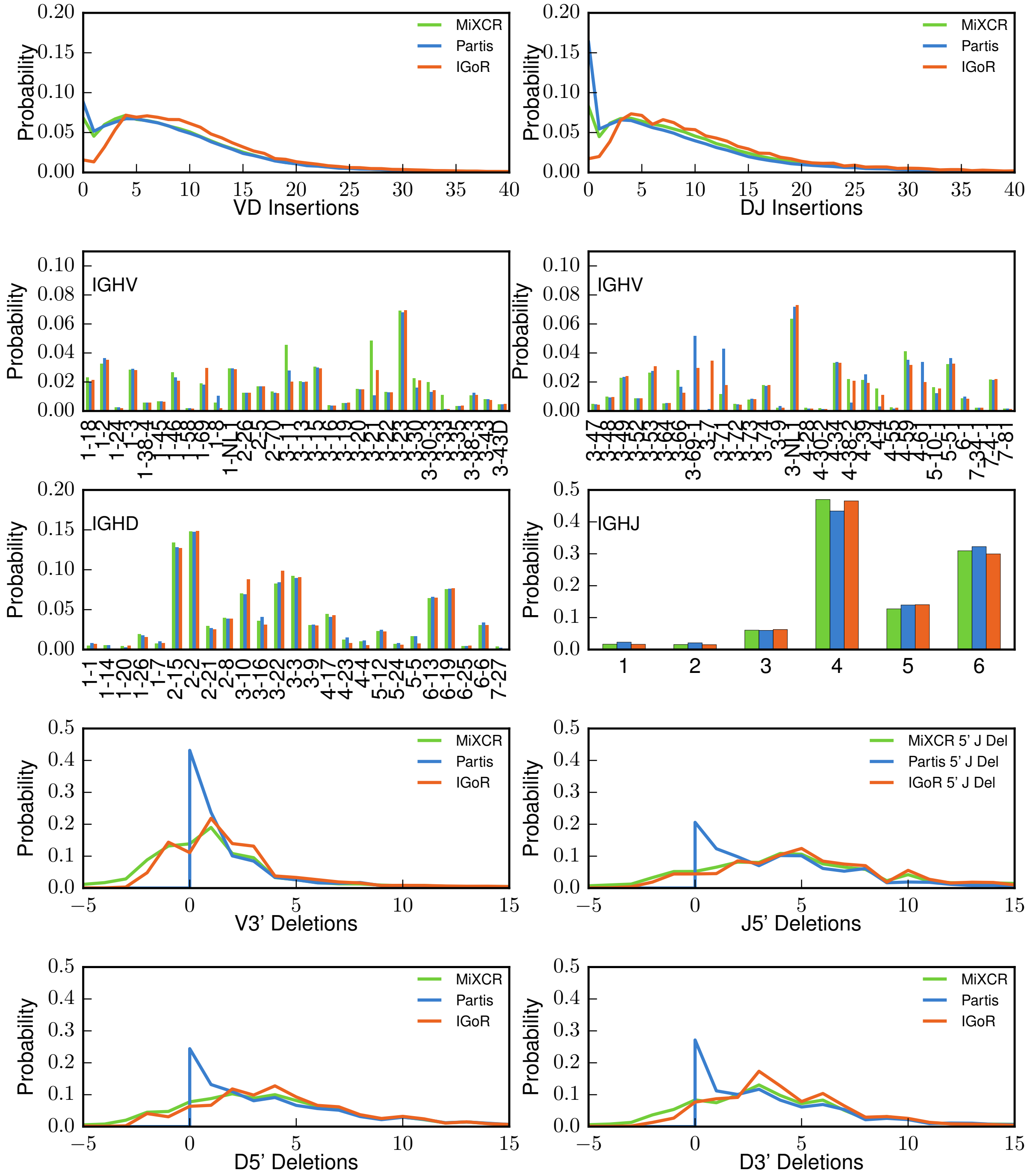

Supplementary Figure 13: **Comparison of marginal distributions obtained from different softwares for hypermutations-free IGH.** IGoR's distributions are obtained directly by IGoR's inference module from nonproductive sequence data. Partis' and MiXCR distributions are obtained by assigning a scenario to each sequence from the data, and then collecting statistics over all sequences. From the two top panels we observe that Partis and MiXCR overestimate the frequency of low number of non templated insertions. In the four bottom panels, negative number of deletions denote palindromic insertions. We observe that the three methods obtain qualitatively different marginal distributions for the number of deletions.

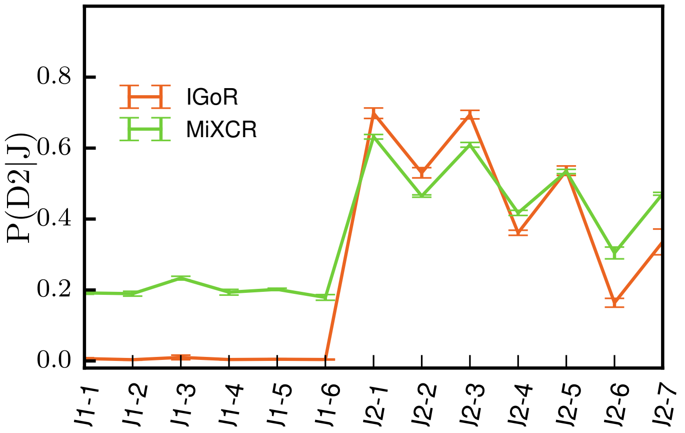

Supplementary Figure 14: **D2-J association in TRB learned from data sequences.** Equivalent of Fig. 4D of the main text, but inferred from the data. Conditional probability of D2 usage, $P(D2|J)$, obtained from real 100bp TCR mRNA data using IGoR and MiXCR. IGoR captures the physiological exclusion between D2 and J1 while MiXCR does not.

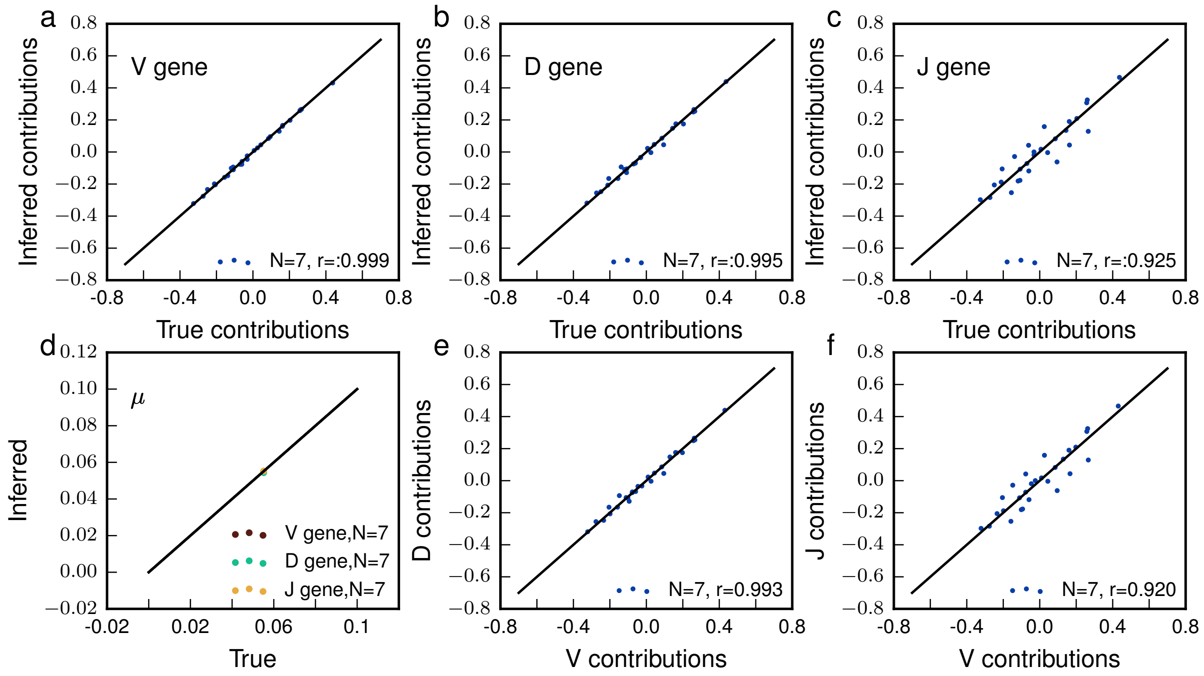

Supplementary Figure 15: **Inference of the 7mer additive hypermutation model from synthetic sequences.** In order to assess the validity of our method we generate synthetic IGH sequences from a heavy chain model learned on naive data sequences. We then generate Poisson distributed errors on the sequences by simulating mutations at each base pair with a Bernouilli process according to the hypermutation model learned on the V genes of memory IGH sequences. We then cut the sequences in 130bp reads in order to mimic real data sequences, and learn the hypermutation model using IGoR. The model can be perfectly inferred on V and D genes, and fairly well on J genes. The slightly worse performance on J can be explained by the limited number of n-mers sampled by genomic J genes.

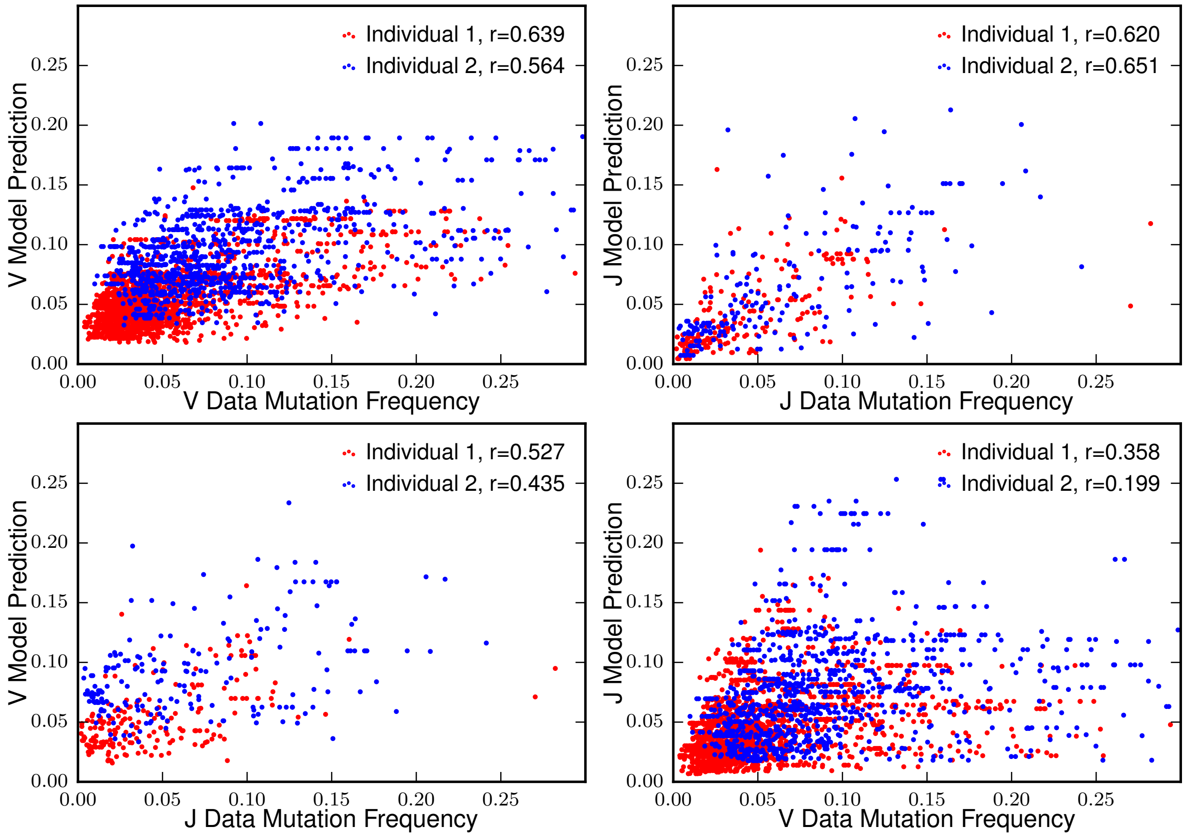

Supplementary Figure 16: **Model prediction of mutation frequencies in nonproductive IGH memory sequences.** The mutation frequency (abscissa) is computed at each position from the posterior probability of a hypermutation at that position given by IGoR, averaged over all sequences for each gene choice. The ordinate represents the mutation probability predicted by the model from the sequence the context at that position in the gene. The two top panels show good predictive power for the gene on which the model was learned. However the two bottom panels suggest that models learned on one gene do not generalize well to other genes, consistent with the different position-weight matrices inferred for each gene (Fig. 18).

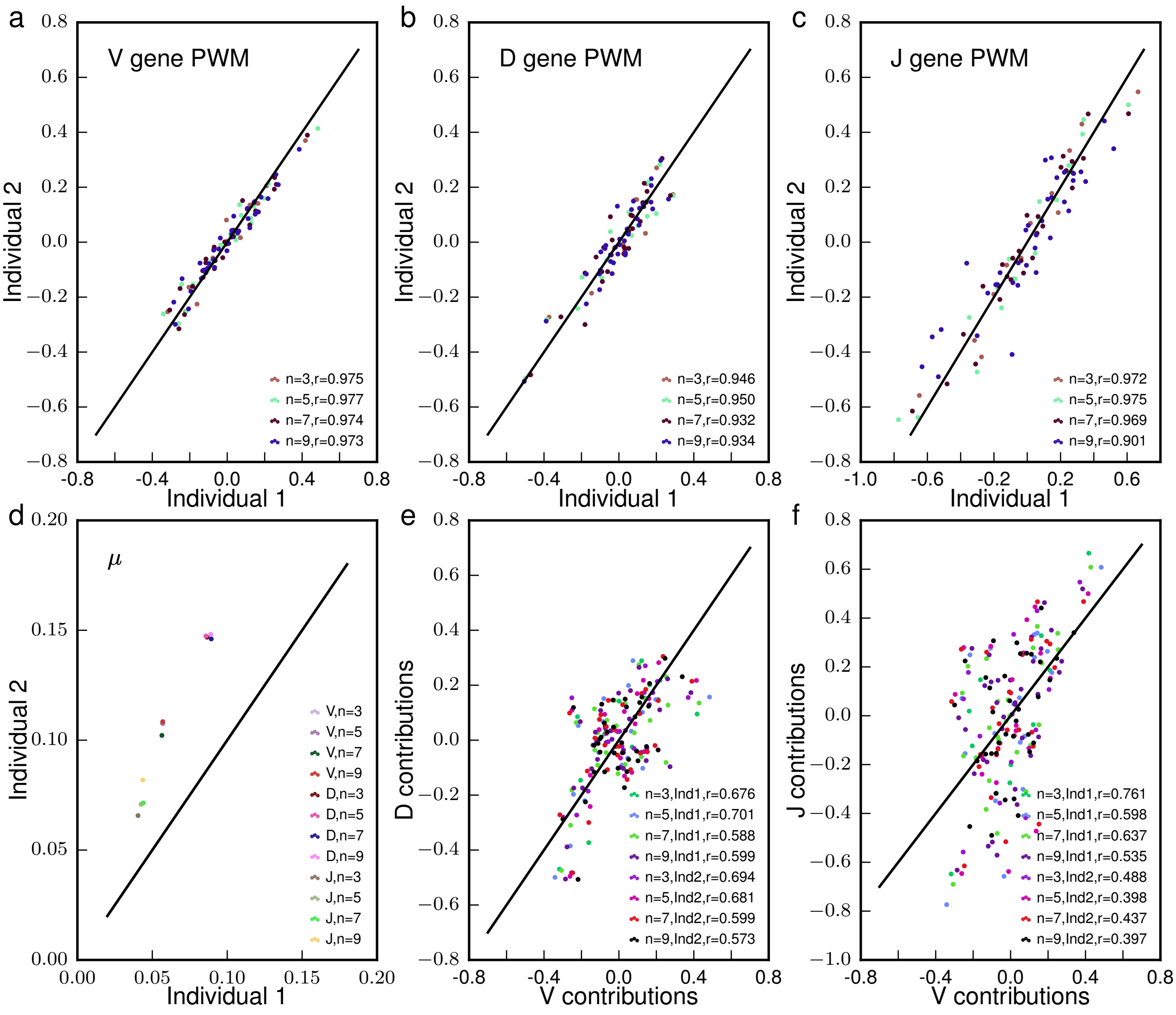

Supplementary Figure 17: **Comparison of the hypermutation models between individuals and between the V, D, and J genes.** **a**, **b** and **c** Comparison of the position weight matrices inferred on the V, D and J genes between the two individuals, for different n-mer lengths. For all sizes and genes the inferred contributions are extremely reproducible from an individual to the other. **d** Comparison of the overall mutational frequency in different individuals and genes for different n-mer sizes. This overall mutational load varies from individual to individual and across genes. **e** and **f** Comparison between matrices inferred on different genes, showing significant differences between the hypermutation models.

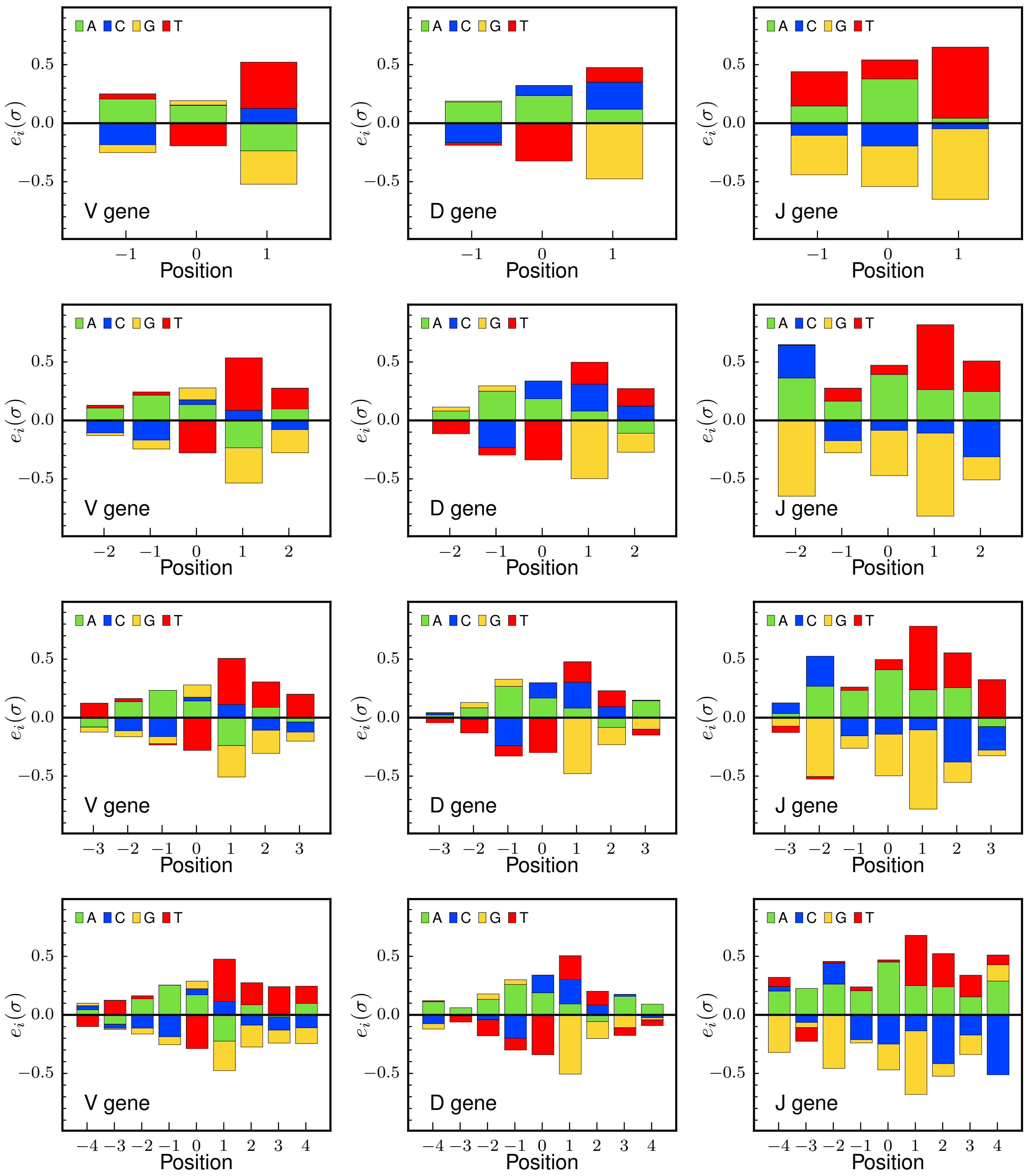

Supplementary Figure 18: **Position-weight matrices for different context sizes, for the V, D, and J genes.** We inferred hypermutation position weight matrices for V, D and J, and for n-mer size $n = 3, 5, 7, 9$. Note that side contributions do not vanish with increasing n-mer sizes.

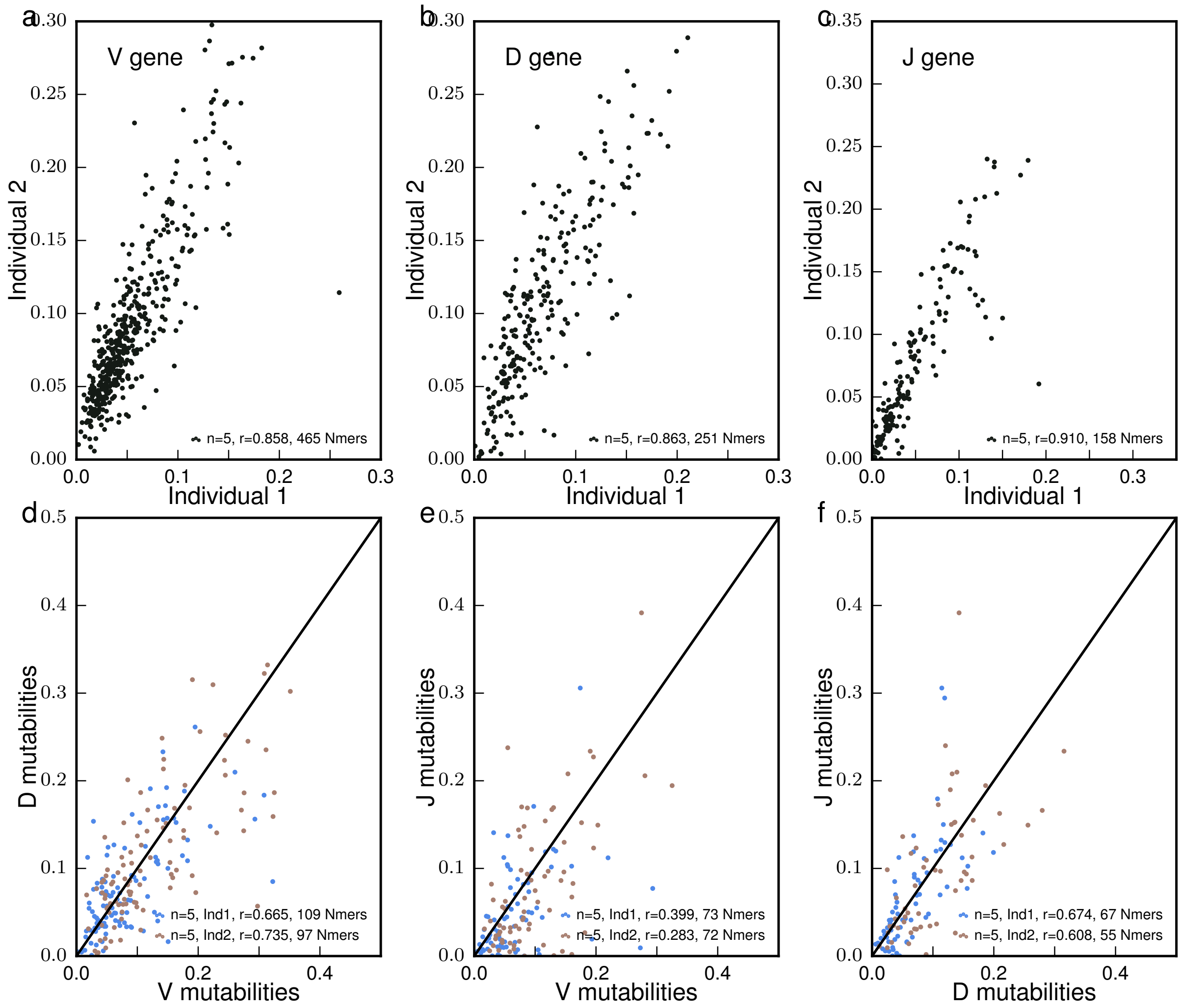

Supplementary Figure 19: **Full 5-mer model of hypermutability**. **a.-c.** Individual-to-individual reproducibility of the infered hypermutation rate for **(a)** V **(b)** D and **(c)** J genes. Each dot corresponds to a 5-mer. Only a fraction of all 1024 possible 5-mers were observed often enough to estimate a hypermutation rate reliably (from 158 to 465 5-mers depending on the gene). **d.-f.** Comparison of the models across different gene families. Agreement between models is reasonable between **(d)** D and V and **(f)** and D and J, but poor between **(e)** V and J.

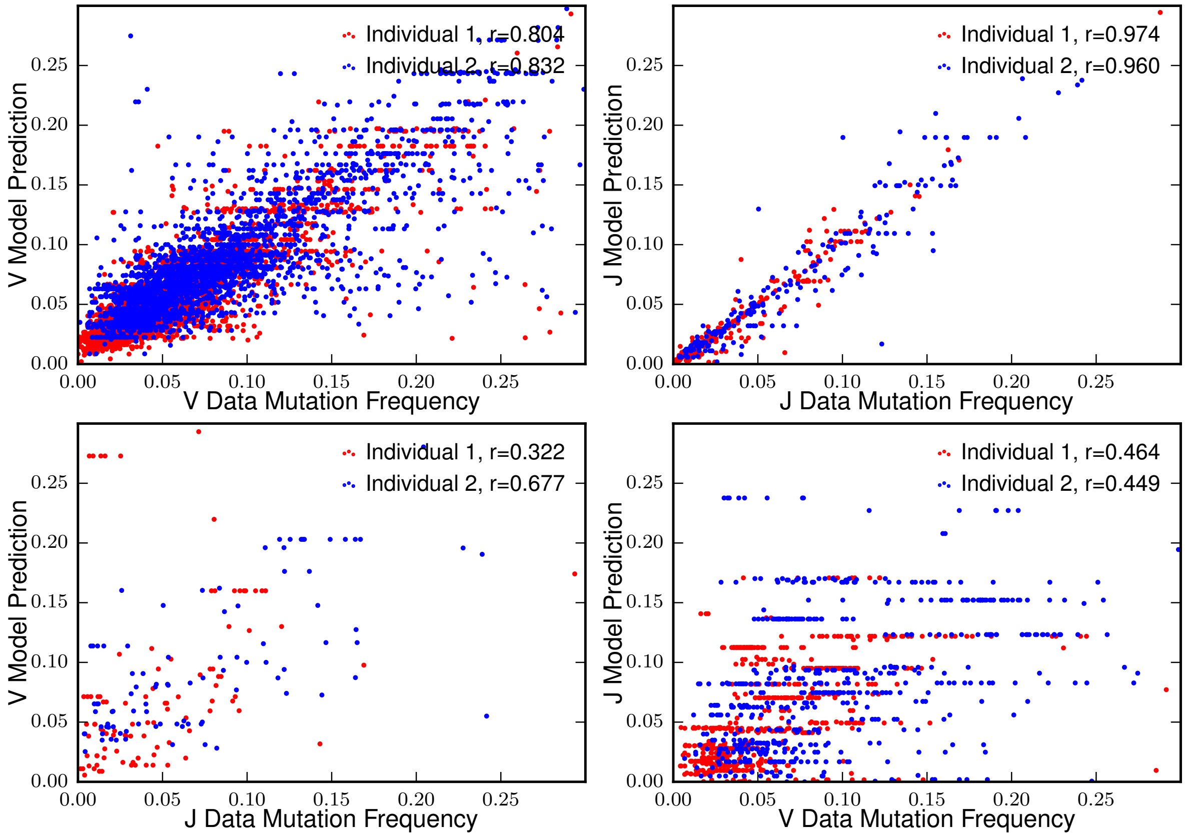

Supplementary Figure 20: **Performance of the full 5-mer model**. Same as Fig. 16, but for the full nonadditive 5-mer model.

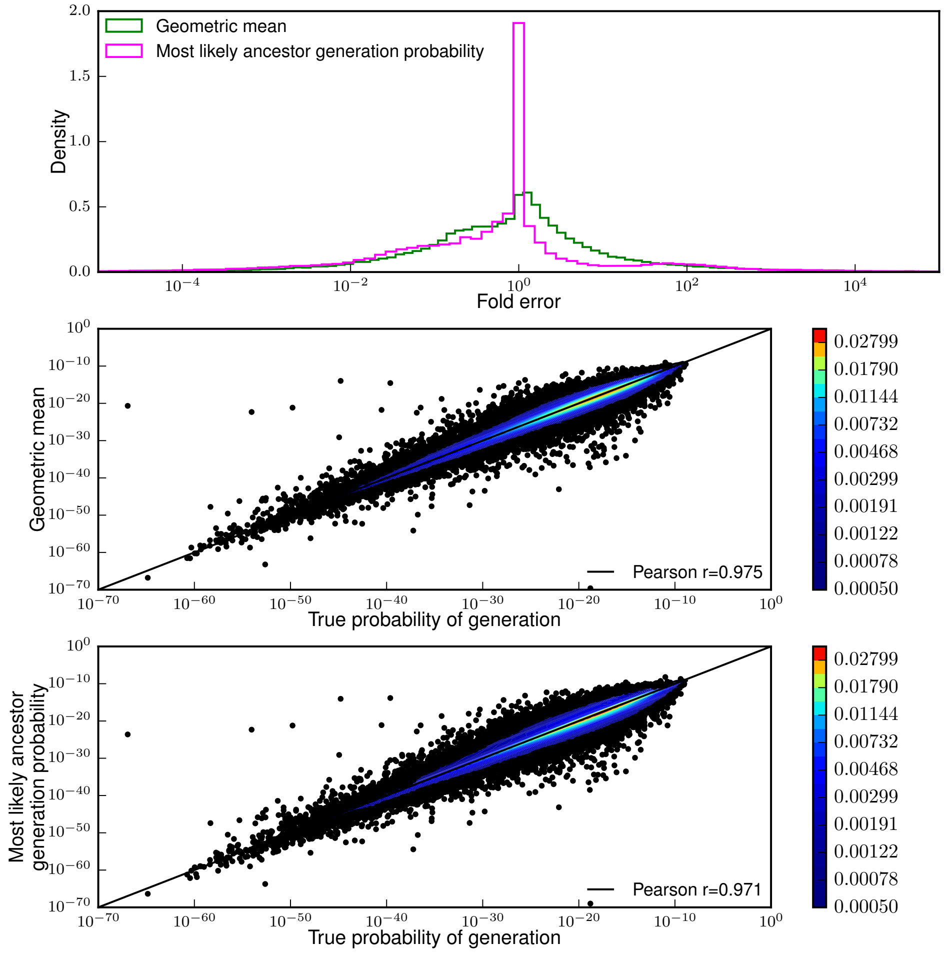

Supplementary Figure 21: **Estimation of the generation probability of synthetic mutated IGH sequences.** We generated synthetic 130bp IGH sequences with IGoR using data-inferred recombination parameters, and calculated their true generation probabilities P_{gen}. We then introduced mutations with a context-dependent rate mimicking hypermutations with an average rate of 5%, and asked whether we could estimate P_{gen} of the unmutated ancestor sequence from the mutated sequence, using two estimators described in SI Text (Sec. 4): the geometric mean of P_{gen} over all possible unmutated sequences weighted by the posterior probability; or the generation probability of the sequence that maximizes that posterior. The middle and bottom panels shows the performance of each estimator for a large number of synthetic sequences (the color map represents the density of sequences). The top panel shows the distribution of fold errors in P_{gen} for both estimators across sequences.

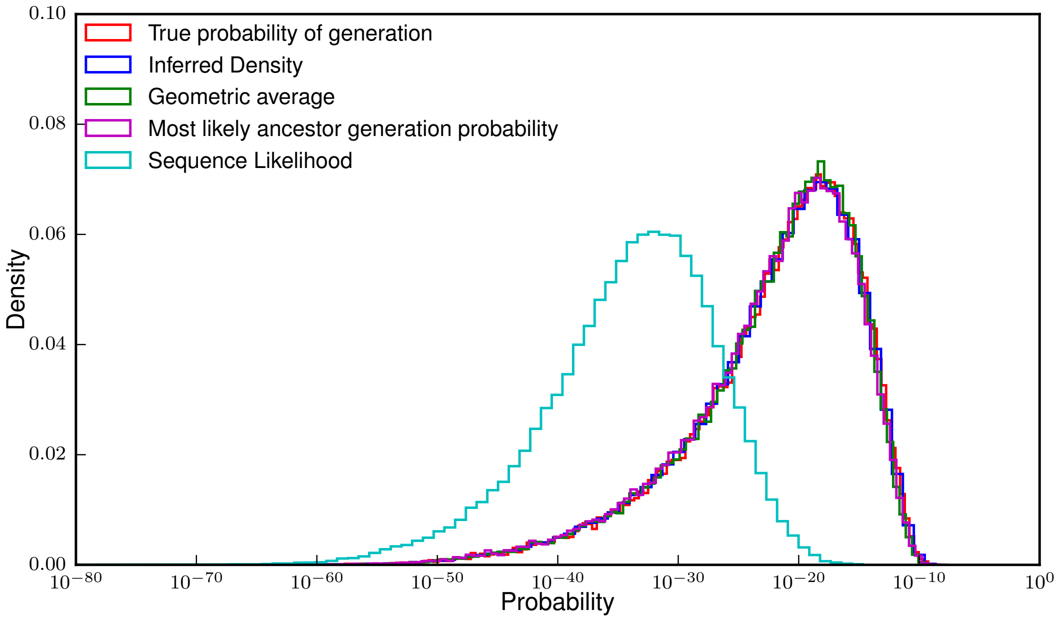

Supplementary Figure 22: **Reconstructed distributions of $P_{\rm gen}$ of synthetic hypermutated IGH sequences**. Distribution of the generation probability $P_{\rm gen}$ obtained by the two different estimators (see 21 for definitions), compared to the true distribution. The "inferred density" (blue) is obtained by pooling together the posterior distributions of $P_{\rm gen}$ of all sequences. The "sequence likelihood" corresponds to the likelihood of the mutated sequences, which differs significantly from the likelihood $P_{\rm gen}$ of their unmutated ancestors.

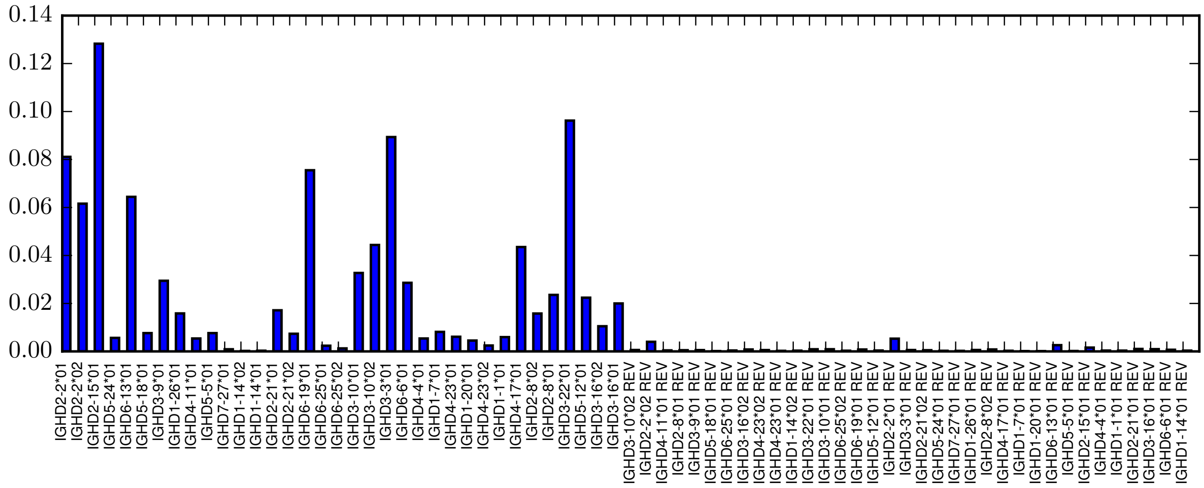

Supplementary Figure 23: **IGH reverse-complement D usage.** We ran IGoR's inference module on non-productive naive IGH sequences again, after adding to the list of candidate germline D segments the reverse-complement versions of these segments (REV in the x-axis). Reverse-complement segment usage is inferred to be negligible, indicating that such recombination events do not occur.

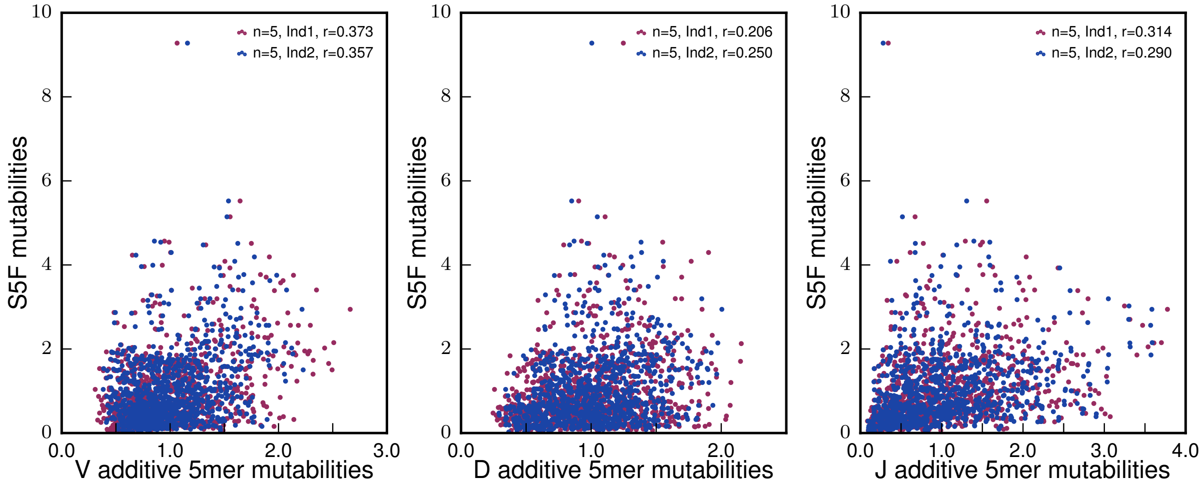

Supplementary Figure 24: **Comparison of the additive hypermutation model with the the S5F model from [13].** The mutabilities (in arbitrary units) of each of the possible 5 mers were compared between the S5F model and IGoR's additive hypermutation model, for each of the V, D, and J genes, and for each individual.

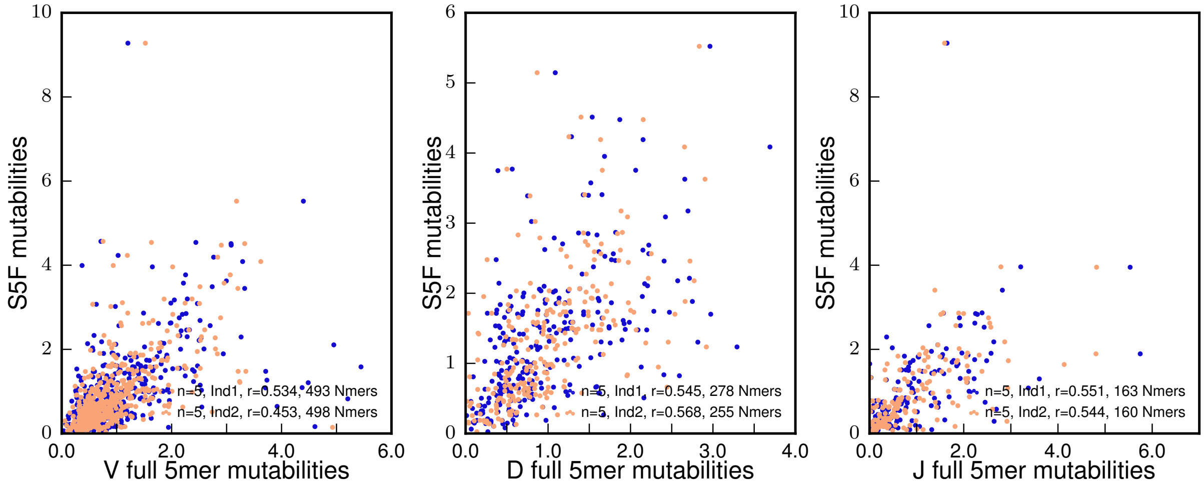

Supplementary Figure 25: **Comparison of the complete hypermutation model with the the S5F model from [13].** Same as Fig. 24, but for IGoR's nonadditive 5-mer model instead of the additive model.

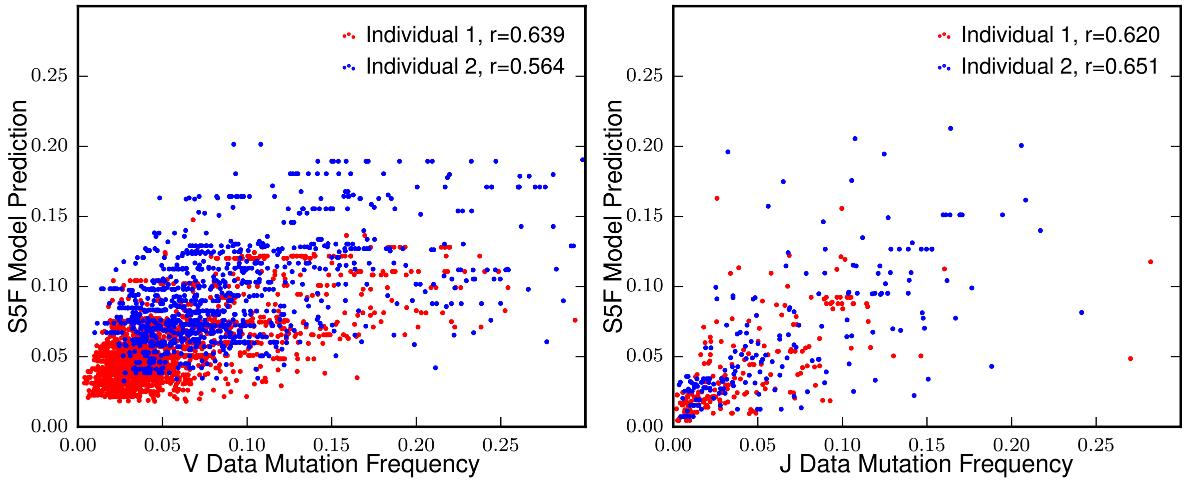

Supplementary Figure 26: **Performance of the S5F hypermutation model from [13] on the sequences analyzed by IGoR.** Same as Fig. 16 and 20, with the S5F model prediction instead of IGoR's model prediction.

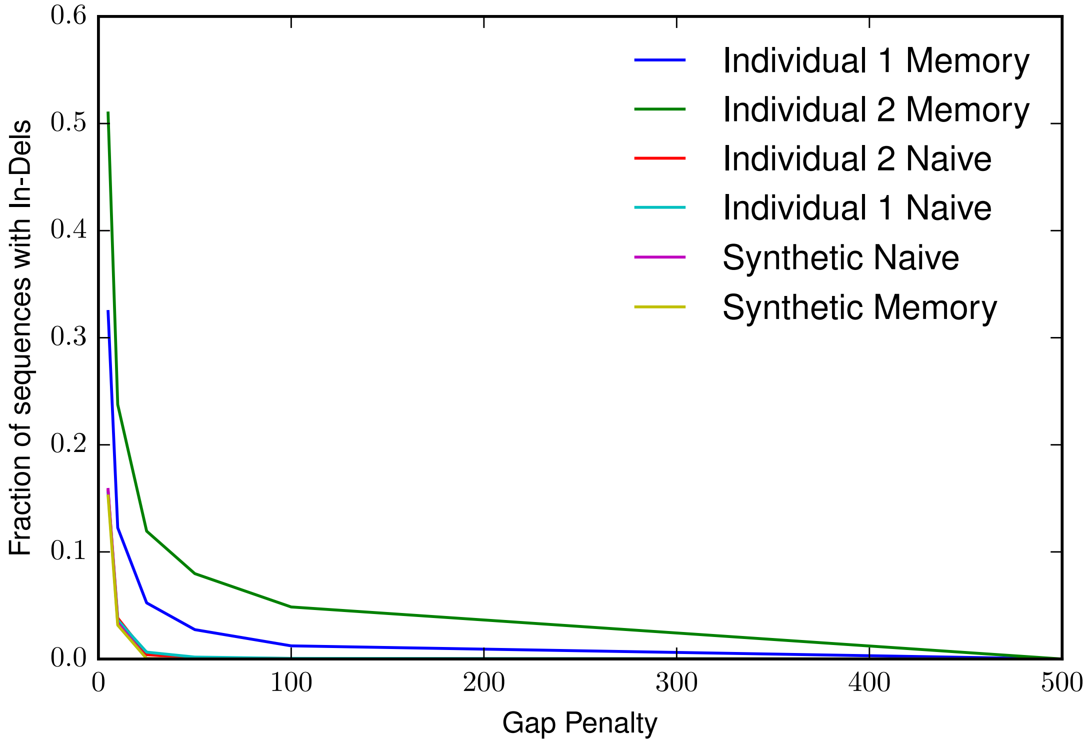

Supplementary Figure 27: **Hypermutation indels.** Fraction of indels identified in the V region among nonproductive sequences (i.e. with a frameshift in the CDR3), as a function of the gap penalty used in the alignment algorithm, for naive, indel-free synthetic, and memory sequences. The naive and synthetic sequences have similar number of detected indels, indicating that naive cells have virtually no indels. The difference between the curves obtained from the naive and synthetic sequences and those obtained from the memory sequences allows us to estimate the frequency of indels to range from 5 to 12%, depending on the individual.

Gene	Gene frequency	RepgenHMM D_{KL}	IGoR D_{KL}
TRAV19*01	0.0782225	0.108	0.00572
TRAV13-1*01	0.0654412	0.0144	0.00419
TRAV13-1*02	0.0628822	0.0190	0.00583
TRAV27*01	0.0551092	0.0212	0.00807
TRAV13-2*01	0.0497209	0.0218	0.0126
TRAV4*01	0.0423985	0.0673	0.00652
TRAV38-2/DV8*01	0.0331126	0.0196	0.00862
TRAV21*01	0.0321689	0.0388	0.0141
TRAV29/DV5*01	0.0317682	0.0611	0.00734
TRAV17*01	0.0300893	0.0434	0.00703

Supplementary Table I: **Comparison of inference accuracy with repgenHMM.** Accuracy of the inference of the distribution of numbers of deletions in the 10 most common V segments (sorted by decreasing frequency), measured by the Kullback-Leibler divergence (in bits). Synthetic data was generated using two distinct distributions of insertions (the same peaked distribution as observed in the real data, and a geometric distribution), randomly assigned to each choice of the V gene. In repgenHMM, the distribution of insertion is assumed to be gene-independent, while IGoR allows for gene-dependent inference of the distribution of insertions. RepgenHMM not only cannot infer the gene-dependent insertion distribution by construction (not shown), but it also infers the gene-dependent deletion distribution much worse than IGoR, as shown here.

---

[1] Murugan A, Mora T, Walczak AM, Callan CG (2012) Statistical inference of the generation probability of T-cell receptors from sequence repertoires. *Proc. Natl. Acad. Sci.* 109:16161–16166.

[2] Elhanati Y, et al. (2015) Inferring processes underlying B-cell repertoire diversity. *Phil. Trans. R. Soc. B* 370:20140243.

[3] Pogorelyy MV, et al. (2017) Persisting fetal clonotypes influence the structure and overlap of adult human t cell receptor repertoires. *PLOS Computational Biology* 13:1–18.

[4] Shannon C (1948) A mathematical theory of communication, bell system technical journal 27: 379-423 and 623–656. *Mathematical Reviews (MathSciNet): MR10, 133e.*

[5] Cover TM, Thomas JA (2012) *Elements of information theory* (John Wiley & Sons).

[6] Elhanati Y, Marcou Q, Mora T, Walczak AM (2016) repgenhmm: a dynamic programming tool to infer the rules of immune receptor generation from sequence data. *Bioinformatics* 32:1943–1951.

[7] Lefranc MP, et al. (2009) Imgt®, the international immunogenetics information system®. *Nucleic acids research* 37:D1006–D1012.

[8] Smith TF, Waterman MS (1981) Identification of Common Molecular Subsequences. *J. Mol. Biol.* 147:195–197.

[9] Neal RM, Hinton GE (1998) in *Learning in graphical models* (Springer), pp 355–368.

[10] Bolotin DA, et al. (2015) Mixcr: software for comprehensive adaptive immunity profiling. *Nature methods* 12:380–381.

[11] Ralph DK, Matsen FA (2016) Consistency of VDJ Rearrangement and Substitution Parameters Enables Accurate B Cell Receptor Sequence Annotation. *PLOS Computational Biology* 12:e1004409.

[12] Robins HS, et al. (2009) Comprehensive assessment of t-cell receptor β-chain diversity in $\alpha\beta$ t cells. *Blood* 114:4099–4107.

[13] Yaari G, et al. (2013) Models of Somatic Hypermutation Targeting and Substitution Based on Synonymous Mutations from High-Throughput Immunoglobulin Sequencing Data. *Frontiers in Immunology* 4:358.
